# Introducing CACIE: Development of the first Conceptual Assessment of Children’s Ideas about Evolution

**DOI:** 10.1371/journal.pone.0331380

**Published:** 2025-09-03

**Authors:** Isabell K. Adler, Daniela Fiedler, Andrew Shtulman, Ute Harms

**Affiliations:** 1 Department of Biology Education, IPN – Leibniz Institute for Science and Mathematics Education, Kiel, Schleswig-Holstein, Germany; 2 Department of Psychology, Occidental College, Los Angeles, California, United States of America; University of Tartu, ESTONIA

## Abstract

The theory of evolution is the core theory of the life sciences. However, due to its counterintuitive nature, learners of all ages have difficulties building coherent knowledge about evolution. Researchers propose to facilitate learning about evolution in school by introducing the topic to children at a younger age to foster learners’ pre-scientific ideas and prevent the establishment of inaccurate beliefs. However, assessment tools that could be used with young children are still lacking. This article presents the development and psychometric evaluation of the interview-based Conceptual Assessment of Children’s Ideas about Evolution (CACIE). The CACIE comprises 20 items about 10 concepts of the evolutionary principles variation, inheritance, and selection. They can be used with six different animal and plant species. The CACIE was tested with 85 children (1) in cross-sectional interviews and (2) in a test-retest design (*n* = 14). The instrument was developed using an empirically validated theoretical framework, informed by published instruments and interviews, and refined through pilot studies and observations. The assessment showed good agreement between raters and moderate test-retest reliability. The validity evidence for the responses generated by the CACIE is discussed, and guidelines for its use to measure children’s ideas about evolution are provided.

## Introduction

The theory of evolution is the core theory of the life sciences. This theory, first detailed by Charles Darwin in his 1859 book *On the Origin of Species*, provides a scientific explanation for why organisms appear well adapted to their various environments, how all organisms on Earth today descend from a single common ancestor, and how the modern biodiversity of life came to be through natural selection [1,2]. Natural selection results whenever populations of organisms vary in their heritable traits and those variations make them more or less likely to survive and reproduce. These circumstances increase the probability of organisms with more advantageous (more “fit”) traits to have more offspring, leading to an increase in frequency of organisms with those traits [3,4]. Modern evidence from paleontology, comparative anatomy, and genetics strongly support the theory of evolution, and genetics, molecular biology, and evolutionary developmental biology have since provided a host of mechanisms by which heritable variations (e.g., new mutations) arise and gets passed on across generations [1].

For humans, these processes are counterintuitive because intuitive explanations and predictions of environmental phenomena shaped by universal cognitive biases cannot be used to explain evolutionary change appropriately – biases such as essentialism (i.e., the assumption that members of a species share an unchanging essence), teleology (i.e., the assumption that traits evolve for a purpose or toward a goal), intentionality (i.e., the assumption that evolutionary change occurs because organisms want or try to change), and anthropomorphism (i.e., the attribution of human characteristics to non-human organisms or processes) [5]. This results in learners of all ages having difficulties building coherent knowledge about evolution (e.g., [6–8]). Therefore, much research has investigated (1) ideas that learners hold about evolution (e.g., *for students*: [8–10]; *for university students and pre-service teachers:* [11,12]), (2) factors that might impede learning about the topic (e.g., *cognitive biases*: [13–16]; *obstacles inherent to the subject:* [17–19]; *curricula and materials:* [20,21]; *political aspects and teacher’s attitudes:* [22,23]), and (3) ways to enhance conceptual knowledge about evolution (e.g., *through teaching practices:* [24–27]; *curricula and materials:* [28,29]; *teacher preparation*: [30,31]; *citizen science (i.e., research involving both professional and non-professional scientists):* [32]).

Researchers have also aimed to facilitate learning about evolution in school by introducing the topic to children at a younger age to foster learners’ pre-scientific ideas and prevent the establishment of inaccurate beliefs that emerge through cognitive biases [29,33–36]. Consequently, for the past two decades, there has not only been a rise in the publication of children’s literature about evolution (see [37]) but also of scientific studies that aim at promoting children’s knowledge of evolution [38]. While those empirical studies provide evidence about their effectiveness of children’s conceptual knowledge, most of the children’s educational literature remains without empirical evaluations. The testing of such material could further improve the quality of early science education [39]. However, to date, there is still a lack of assessments to do so.

The use of assessment tools is a standard practice in cognitive psychology as well as in science education research, helping to investigate pre-existing ideas, explore cognitive relationships, monitor learning progress or test the effectiveness of interventions and pedagogical practices [40,41]. In science education, standardized assessments for kindergarten children mostly focus on general scientific literacy and are norm-referenced, meaning they allow for the comparison of an individual’s results with those of a normative reference group. In general, they are used to evaluate children’s school readiness, monitor their developmental progress, or assess achievement of mandated academic standards [39,42]. While assessments for school students usually include paper-pencil tests, assessments of kindergarten children need to account for their different pre-conditions. Therefore, they typically rely on either observation by educators or researchers (e.g., [43]) or on individually administered evaluations (e.g., [44]). Individually administered assessments need to present the content in a developmentally appropriate manner by using familiar contexts and vocabulary or illustrate the question or responses (e.g., in form of explanatory pictures, videos, or physical objects). For instance, the *Science Learning Assessment* (SLA) measures children’s conceptual knowledge of the nature of science as well as concepts of the living and physical world through 24 items [44]. The assessment is designed as a multiple-choice test with each response option being represented through an illustration, one illustration representing the right answer. The children are not required to talk but only need to point at one of the three illustrations. The *Preschool Science Assessment* (PSA) covers concepts of the life, Earth and space, physical and energy sciences as well as science practices [39]. The set of 80 items are provided in form of a flipbook including instructions for the examiner as well as pictures or manipulatives, like measuring squares, for the children who have to answer either verbally or point, sort, sequence, or measure [39]. The *Science-K Inventory* consists of 30 items about experimentation, data interpretation, and the nature of science [45]. Again, the question format is a multiple-choice format illustrated through pictures with one correct answer and two distractors. However, assessments for more specific science topics are mostly lacking. For instance, instruments used in evolution education research are often not suitable for children because they test declarative knowledge, utilize scientific terminology and require proficiency in reading and writing (see also [41,46]). Although researchers have developed a repertoire of more than two dozen assessment tools targeting the topic of evolution (concept inventories; see [47]; e.g., [48–50]), assessment tools for younger, pre-literate children are lacking in this field. As a consequence, studies with young children mostly rely on self-developed, non-validated interview questions (for an overview see [38]), making comparison between studies less reliable ([51]; for an overview see [52]). A notable effort has already been made by Sá-Pinto and colleagues [53], who were the first to develop an evaluation framework for pre- and posttests on elementary children’s understanding of evolution by natural selection. The children are presented with a selection scenario (i.e., a butterfly population with different morphological traits is introduced to an island with different resources) and are tasked to make a prediction about the evolution of the population. The test was designed for fourth graders and comprised a writing and drawing task as well as an individual interview.

Therefore, our aim was to lay the groundwork for a standardized instrument designed to assess kindergarten children’s ideas about evolution. In this article, we provide our interview-based Conceptual Assessment of Children’s Ideas about Evolution (CACIE) by describing the development process and evidence of its validity. It should be noted here that we aimed to standardize the CACIE in the sense that identical test materials are presented to all test takers, administration procedures are strictly followed, and prescribed scoring rules are applied consistently (see [51]). However, it should be noted that, in its current state, it does not allow for direct comparisons between test-takers and a normative group.

## Methods

### Development of the CACIE

The CACIE is the culmination of five years of research by the [name deleted to maintain the integrity of the review process] project (Table 1). Our methodology began with a rigorous systematic literature review of interview and intervention studies of the past two decades that assess children’s evolution understanding.

**Table 1:** Overview of the multistep process in developing the CACIE.

Year	Steps in development	Sample size	Publication
2019	Literature review		[38]
2021	Development of interview questions and graphics		
	Piloting (age 5–6)	*N* = 9	
	Definition of categories		
2022	Data collection (age 5–6)	*N* = 24	
	Analysis of the initial data		[54]
	Revision and digitization		
	Piloting (age 5–8)	*N* = 3	
	Cross-sectional interviews (age 5–6)	*N* = 15	
	Cross-sectional interviews (age 7–8)^a^	*N* = 19	
2023	Cross-sectional interviews (age 5–6)	*N* = 37	
	Test-retest analysis (age 5–6)	*N* = 14	
	Final analyses		this publication

^a^This sample of older children served to analyze demographic differences.

Based on this review and the review of assessments for older target groups [47], we developed an initial interview prototype consisting of 33 questions about evolution (see Chapter Key concepts being tested). An expert in child development assisted us in ensuring that our questions used age-appropriate speech. The prototype was refined iteratively in three pilot testings with three children aged 5–6 years in each round. This data served to define the category system for qualitative content analysis [55]. Subsequently, the first interview version was administered to 24 children. The interviews were audio-recorded, transcribed, and analyzed through qualitative content analysis. The findings of these initial interviews were summarized in another article [54].

Based on this first implementation and in line with the theoretical framework, we revised and streamlined the items and the category system, resulting in a final set of 20 items (see Chapter Format and item design). A researcher with experience in concept inventories for evolution helped us to review our items to assess their accuracy and relevance in addressing our suggested concepts (see Chapter Key concepts being tested). For convenient use, we digitized the survey and implemented it in a survey platform, enabling us to categorize answers during the interview without having to rely on audio-recorded data. To make our interview tool more widely available, we prepared the survey in two languages (English and German). The digitized version was piloted with three children in the United States, and after a final revision, we conducted interviews with 15 children aged 5–6 years and 19 children aged 7–8 years in the United States*.* This final version was also tested in a test-retest design with 14 children aged 5–6 years in Germany.

### Key concepts being tested

The CACIE was developed based on a theoretical framework that includes ten key concepts of the evolutionary principles of variation, inheritance, and selection, which is a widely used framework in evolution education research (e.g., [50,53,56–60]). For each key concept, we identified two essential components (i.e., subconcepts) that have been examined in evolution education research (Table 2).

**Table 2:** Overview of the assessed principles with their key concepts and subconcepts.

Principle	Key Concept		Item	Subconcepts
Variation	Individual Variation		V1A	Variation in heritable traits
			V1B	Variation in “inner” non-visible traits
	Origin of Variation		V2A	Between-parent variation
			V2B	Within-parent variation
	Differences in Fitness		V3A	Variation in beneficial traits
			V3B	Effect of beneficial traits on longevity
Inheritance	Reproduction		I1A	Biological parents/ Sexual reproduction
			I1B	Hyperfecundity and population size
	Inheritance of Variation		I2A	Resemblance in families
			I2B	Variation between siblings
Selection	Limited Resources		S1A	Limited resources in the environment
			S1B	Different distribution of resources between members of a species
	Differences in Reproduction and Survival Rate		S2A	Different survival rates within a population due to different traits
			S2B	Different reproduction rates within a population due to different traits
	Changes in Population		S3A	Change in trait frequency after obvious disadvantage
			S3B	Change in trait frequency after implicit advantage
	Speciation	Origin and extinction of species on Earth	S4A	Origin of species
			S4B	Extinction of species
		Common Ancestry	S4C	Families and phylogeny
			S4D	Species boundaries

#### Variation.

Variation is the prerequisite for natural selection. Thus, it is an essential concept to understand natural selection and overcome essentialist biases [61–63]. Variation can be described by the key concepts *individual variation*, *origin of variation*, and *differential fitness*. Individual variation (also referred to as within-species variation) describes the phenomenon that all individuals are inherently different [64]. In contrast, between species variation would refer to how members of different species vary. Thus, understanding variation is a combination of appreciating the similarities that members of a species share but also being aware of the individuality of each species member. The origin of variation is often attributed to random genetic mutations, larger-scale chromosomal rearrangements, or (in the case of sexual reproduction) recombination during meiosis, among other mechanisms [65]. Most genetic changes are detrimental or do not lead to a change in the phenotype or differences in fitness, meaning beneficial and unbeneficial traits that affect an individual’s ability to cope in the environment (neutral theory of evolution; [66]). There are contradictory findings about whether children have a high [67] or low acceptance of within-species variation [68]. Regardless of children’s baseline levels of acceptance, this research shows that essentialist beliefs can be reinforced by using generic language or emphasizing the benefits of a trait [68,69]. Moreover, older children tend to have a better understanding of variation in animals [67,69,70]. In contrast, understanding the origin of variation is more difficult for children as it requires knowledge of genetics and inheritance [70].

#### Inheritance.

The principle of inheritance describes (1) how new individuals arise from the genetic material from one parent through cloning of gametes or two parents through the union of male and female gametes, (2) that individuals produce more offspring than would be necessary to sustain the population size (hyperfecundity), and (3) that sexual reproduction and inheritance result in offspring showing variation. Aspects (1) and (2) can be subsumed under *reproduction* and (3) referred to as *inherited variation*. Children usually develop their initial understanding of reproduction and inherited variation through their family. Thus, it is not surprising that children primarily view families as social constructs rather than biologically related units. Consequently, the involvement of two parents is primarily attributed to social factors rather than seen as a necessity of sexual reproduction [71]. Still, children at kindergarten age seem to have a rudimentary idea of inheritance [72–74] but are sometimes biased toward one parent (mostly the mother) being more strongly responsible for the offspring’s traits (i.e., mother bias) or they reason upon information irrelevant for inheritance (e.g., social proximity or parents’ preferences; [75,76]). Another common belief that has been observed to be robust to intervention and to persist into adulthood is that offspring tend to exhibit a stronger resemblance to their same-sex parent (i.e., sex-matching; [77,78]). Not much research has been done on children’s ideas about animal and plant reproduction outside the context of heritable traits. However, evidence indicates that children and students have less knowledge and less accurate ideas about plant compared to animal reproduction [79–82].

#### Selection.

The principle of selection describes how *limited resources* in an environment lead to individuals within a population having *different rates in survival and reproduction*. Such differences result in changes in the frequency of traits in populations (*changes in populations*) that further impact survival and reproduction. Over time, these changes can cause a population to diverge from its original gene pool and phenotype to the extent that it can then be considered a new species (*speciation*). Thus, the process of speciation explains how two modern species can be related to each other, descending from a common ancestor that lived a long time ago. When considering changes in populations, children often use developmental, transformationist or teleological arguments [83–85]. Children’s reasoning about selection appears to be sensitive to speech, with anthropomorphic and teleological explanations influencing their understanding of differential survival and reproduction [86]. Further, contextualizing differences between individuals and the impact of those differences can help children understand differential survival [67]. However, evidence suggests that children in second grade and above are better capable of comprehending selection than young children [29,84–90], who show difficulties explaining natural selection and imagining a time when certain animals did not exist [84,91,92]. Concerning the origin of species, elementary school children have been shown to hold creationist, essentialist, spontaneous generationist or Lamarckian beliefs [87,91,93–96]. Still, young children can benefit from interventions with simplified representations of, for instance, extinction, homology or adaptations [90,93,95,97–99].

### Organizational level and order of items

The interview items address different organizational levels (i.e., individual, population, species), and were arranged in a logical order with an ascending degree of complexity (from one individual to two individuals to populations and species). Consequently, the items regarding variation (V1, V2, V3) and inheritance (I1, I2) as well as the key concepts limited resources (S1) and origin and extinction of species (S4A, B) were placed in the first section (Table 2). The selection questions that entail thinking in terms of populations and considering effects of environmental factors (S2, S3, S4C, D) were contextualized through a short story, referred to as the selection scenario (see 66), and were situated in the second section.

### Biological examples used in the CACIE

While assessments targeting adolescents and adults normally use real-world examples (e.g., [48–50,100]), many assessments that target young children often use fictitious examples [53,69,89,92]. The use of fictional rather than familiar examples has the advantage that children are less affected by prior knowledge. However, it also requires children to distinguish between realistic and fictional features and to evaluate whether they should apply real causal knowledge or imagination [101]. Hence, we decided to use real-world examples.

In addition, most assessments for children rely on animal examples exclusively. Since context factors, such as the biological kingdom, have been shown to influence students’ and children’s responses [58,102,103] we chose an equal amount of plant and animal examples to provide a balanced amount of examples between the two conditions (i.e., the plant and the animal condition). Furthermore, we selected species that belong to different folk biological categories (e.g., both vertebrates and invertebrates in the case of animals and ferns, flowers and trees in the case of plants; see Table 3).

**Table 3:** Overview of the chosen examples.

Kingdom	Animals			Plants		
**(Folk) Biological Categories**	Invertebrates	Vertebrates		Polypodiophyta	Flowering plants	
	Snail	Bird	Mammal	Fern	Flower	Tree
**Species**	Brown-lipped snail	Hooded crow	Red fox	Eagle fern	Dandelion	Apple tree
	*Cepaea nemoralis*	*Corvus cornix*	*Vulpes vulpes*	*Pteridium aquilinum*	*Taraxacum officinale*	*Malus domestica*

### Format and item design

**Fig 1:**
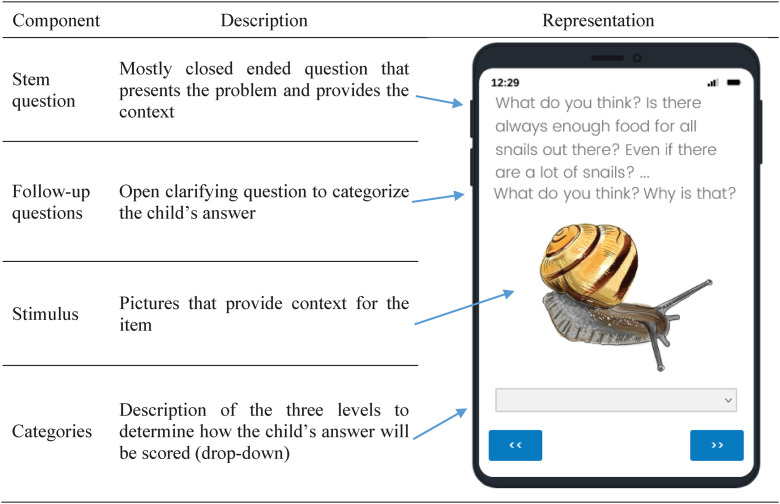
Structure of the CACIE items.

The CACIE targets kindergarten children, who probably (1) are not yet literate, (2) differ in their linguistic development, and (3) do not yet have declarative knowledge about evolution [104]. To account for these different pre-conditions, we chose an interview format in which the participants give answers to both open and closed questions (Fig 1). Our items are standardized in layout and terminology to ensure consistency throughout the assessment. Every item has a visual stimulus for the children and starts with a stem question followed by follow-up questions that enable the interviewer and interrater (i.e., an additional evaluator who independently applied the same coding scheme as the interviewer to verify the reliability of the scoring procedure) to categorize the child’s answer. Most follow-up questions encourage active text production. However, to accommodate the varying language proficiency levels of children, we also provide closed follow-up questions as an alternative in instances where they can be formulated without providing excessive suggestions or introducing new information [105]. Stem and follow-up questions are preceded by the phrase “What do you think?” to create a comfortable environment and emphasize that the questions focus on the children’s ideas rather than their knowledge.

Additionally, realistic drawings of the example species serve as a visual prompt that supports comprehensiveness of the questions by providing additional clarity and context, such as highlighting the relevant structures [106]. To ensure the questions were easily understandable, we simplified the terminology and used short sentences with simple syntax. The use of a simple context or short narrative allows the questions and category system to probe children’s conceptualizations rather than their declarative knowledge. This approach facilitates the communication between the researcher (or interviewer) and the child and thus enables the researcher to better understand the child’s ideas.

The stem questions were either developed or adapted from prior studies that had assessed adults’ or children’s understanding of variation, inheritance, or selection, (e.g., [48–50,67,69,94,107]. In doing so, we were able to draw on the expertise and knowledge of other researchers in the field, increasing the likelihood that the items are valid and reliable. To tailor the questions to our research aims, we employed various techniques, such as adding or reducing aspects that did or did not align with our objectives (see Table 4). Additionally, we made the questions applicable to different examples from the plant and animal kingdoms. For this purpose, we created each item as a template that can be filled in with specific information (e.g., name of the species, anatomical structure, essential resource).

**Table 4:** Examples of adapted items.

Concept	Version	Question
**Variation:** Individual variation (Item V1A)	Emmons & Kelemen, 2015 [69]	See this hergob’s sprogs. The hergob that was found has fuzzier sprogs in its ears. Fuzzier sprogs make it easier to hear danger coming. Do you think all hergobs in the group could have fuzzier sprogs in their ears?
	Adapted version	Look. The [taxon] has [heritable trait]. What do you think? Do all [taxa] have [heritable trait]? ... What do you think? Why is that?
**Inheritance:** Reproduction (Item I1B)	Anderson et al., 2002 (CINS, Item 11) [48]	Assuming ideal conditions with abundant food and space and no predators, what would happen if a pair of guppies were placed in a large pond?
	Adapted version	Imagine. We put a group of [taxa] onto an island by themselves with a lot of [resource] and [resource]. We leave them alone and then check in with it many years later. What do you think? Are there still as many [taxa] as before? Or are there more or less? ... What do you think? Why is that?
**Selection:** Differential survival and reproduction rate (Item S2A)	Kalinowski et al., 2016 (CANS, Item 2) [49]	A disease infects many ant colonies in a forest. The disease does not affect anteaters, but kills most of the ants. What is most likely to happen to the anteaters? a Anteaters will grow slightly longer tongues. b Anteaters will find other food. c Anteaters will share the food available. d Many of the young anteaters will die. e Anteaters will survive on less food.
	Adapted version	Now there is a [disaster] on the island and [change in the environment happens that affects individuals with a certain trait]. What do you think? What happens now? ... What do you think? Do some of the [taxa] die?

### Coding procedure

The participants’ responses get scored on a scale from 0 to 2 with a category system based on the level of scientific accuracy (following the methods of [50,63,108]. Each item addresses a key concept of evolution and, in principle, aims to evaluate (1) whether a child accepts the targeted concept, and (2) whether their reasoning is in line with evolutionary thinking. Responses meeting both criteria are scored as 2, and those meeting neither are scored as 0. A score of 1 is given for ambiguous or partially aligned responses. Thus, the scale reflects a continuum from unscientific (0), to emerging or intermediate (1), to relatively advanced or more accurate ideas (2). The category system includes a description of the three levels for every item. Given the exploratory stage of the CACIE and the lack of comparable tools on evolution for kindergarten children, the definition and application of the scoring criteria involved some degree of interpretive judgment. To ensure that these distinctions nevertheless reflect meaningful differences in children’s ideas about evolution, the development of the items and category system was informed by prior empirical studies (see Description of the items). In addition, the piloting data and the first data collection (see [54]) were used to refine the category descriptions and ensure they realistically align with the expression of children at the kindergarten age. (All items, including the category system, are provided in the Supporting Information S1 File).

### Description of the items

#### Variation.

**Individual Variation:** Following prior studies on individual variation, the CACIE includes items concerning external (V1A) and internal traits (V1B; [68,69]). We selected the color of an inner structure as the internal trait (i.e., white stomach in the animal and white sap in the plant conditions; see [68,69,109]). Unlike these studies, we excluded behavioral traits because we wanted to make sure that the questions could be applied to all organisms (including not just animals, but also plants, fungi, bacteria, and other microorganisms). Behavior in plants is an abstract concept that is more challenging to observe, which makes it difficult to visualize and discuss it with young children.

Explanations are considered more accurate (2) if they acknowledge an inherent variation (naturally present in all individuals). If variation is only attributed to age, gender, environmental factors or invalid factors (see also [67]), the response is scored as 1. When children reject individual variation, we assign 0.

**Origin of Variation**: Given that children likely lack knowledge of genes and genomes (which involve molecular-scale variation; [60,62]), we propose that an age-appropriate understanding of variation can be framed in terms of inter-parental variation (individual-scale variation). The questions are adapted from the study of Gormley and colleagues [67] who examined children’s ideas about variation in frogs within the same family. V2A prompts children to explain why two randomly chosen individuals of the same species display differences in a heritable trait (between-parent variation). V2B addresses children’s comprehension of the idea that parents and their offspring exhibit variation due to differences among those parents (within-parent variation).

The score of 2 indicates an explanation involving inter-parental variation or, in the case that the children would have already learned about molecular-scale variation, other random factors, such as mutations. A 1 indicates a response focusing on age, gender, or environmental factors, and 0 is given to explanations that deny variation, trace variation to between-species variation, or are unsuitable for the question.

**Differences in Fitness**: For the CACIE, we selected traits associated with successful foraging, building upon previous research conducted by Kelemen and colleagues [92]. We present children with a favorable precondition (sense of smell in the animal and long roots in the plant conditions), and asked if every individual of the example species would possess this trait (V3A, [49,68]). We assign a score of 2 if children acknowledge inherent variation in all individuals. Conversely, if variation is attributed solely to factors such as age, gender, environmental influences or invalid factors, the response is scored as 1. Rejecting potential differences in fitness scores 0.

We also ask the children to speculate on the impact of not possessing the beneficial trait, and whether this would impact the longevity of an individual (V3B). Responses indicating that the trait would not affect the individual are scored as 0, while those acknowledging potential negative effects such as limited access to essential resources, but rejecting the notion that this would impact the individual’s longevity, are rated as 1. Responses that recognize both the impact on the individual and the potential earlier death receive a score of 2.

#### Inheritance.

**Reproduction**: The concept of reproduction is assessed through questions about sexual reproduction (I1A; [76]) and hyperfecundity and population size (1B; [48]). When discussing the biological concept of reproduction in animals and plants with children, it is critical to handle the topic with care and sensitivity. The content should not be sexualized, as this may create confusion or discomfort for the children. Therefore, in the CACIE, we use the number of assumed parents as an indicator for the children’s concept of reproduction (IA1). We use family terminology asking the children if the individual of the example species has a mother and/or a father. However, if children do not respond to the family terminology, we clarify if they have a similar concept that uses another terminology by asking them what it would take for an individual to come to life. The interviewer then adapts to the terminology preferred by the child in the follow-up questions. Concerning hyperfecundity (and its effect on population sizes), we ask the children to imagine a group of individuals left alone on an island with sufficient resources and to estimate whether the population size would change with time.

More accurate explanations (2) acknowledge that individuals of the example species can have two parents and that population sizes increase under ideal conditions due to reproduction. The intermediate category (1) is met when children assume one biological parent of the same species or understand a population as a fluid construct, that can increase or decrease by factors other than reproduction (e.g., population grows due to migration, population decrease due to death). If children reject that a member of the same species is involved in the procreation of an individual, or reject that population sizes would change over time, their response is scored 0.

**Inherited Variation**: Inherited variation is assessed through inheritance (I2A) by assessing if children determine potential relatedness through resemblance and inherited variation (I2B) by asking if and why siblings would show variation. In I2A, children are tasked to identify possible parents for an individual and asked to justify their choice [72,110]. This item follows the sexual reproduction item (I1A). It should be adapted when children scored in I1A (i.e., they reject the possibility of two parents) or skipped when children scored 0 (i.e., they reject the involvement of any biological parent) in order to not give them the impression that they answered incorrectly or influence their ideas.

Their response is considered relatively advanced (2) if they use heritable traits to identify possible parents and adhere to the principles of inheritance (e.g., no inheritance across different species). In the case that the child assumes the individuals of the species to only have one biological parent, this should not affect the score of the inherited variation items. If they use heritable traits but violate the logic of inheritance, their explanation is scored as 1. On the other hand, if they attribute family status solely to height, age, or invalid factors, their response receives a score of 0.

In I2B, the children are asked to judge if and why individuals look different than their sibling(s) (see [67]). Following the common misconception that offspring would be an exact copy of their parents [76], siblings would have to look the same. Thus, children that deny variation would score 0. If variation between siblings is explained by environmental factors, age, or gender solely, participants receive a score of 1. More advanced explanations (2) honor recombination by at least mentioning the variation between parents without assuming a gender-based inheritance (i.e., females look like their mothers, males look like their fathers). In cases where a child would refer to identical twin as an explanation for why siblings look alike, the interviewer would have to pose follow-up questions to steer the discussion toward typical sibling variation.

### Selection

**Limited Resources**: To assess the concept of limited resources children are asked whether they believe essential resources in the environment to be infinite or finite (S1A) and to be distributed equally or unequally within a species (S1B; [48]). Children who postulate that resources are unlimited and evenly distributed are given a score of 0. Those who acknowledge that resources are finite or unequally distributed but do not provide a satisfactory explanation receive a score of 1. Children who identify abiotic (such as climate or location) or biotic factors (such as competition) as causes of limited resources and unequal distribution receive a score of 2.

**Differences in Reproduction and Survival Rate**: The concept of differences in reproduction and survival rate in a population is enacted in a short scenario to contextualize the different factors affecting reproduction and survival rate in an age-appropriate manner (see also [53,67]). The children are introduced to a population (in CACIE: hooded crows or eagle ferns) that lives on an island and whose individuals differ in a trait (i.e., crows with long and short beaks that prey on different food sources [beetles or seeds]; ferns with poisonous and nonpoisonous leaves that are preferred or avoided by herbivorous animals). They are then told about an environmental change that affects one of the variants in the population (i.e., beetles die out due to a natural catastrophe; grasshoppers that prey on plants arrive at the island). The children are then asked how the change would impact the individuals and if differences in survival (S2A; [48]) and reproduction (S2B) might appear.

Children score 2 when they expect the affected variants to die and to have fewer offspring. When children either expect the variants to be affected but do not assume that this would affect the survival and reproduction rate, or assume differences in reproduction and survival rates but pick the other variant to be affected, they score 1. Children that reject that the environmental change would affect the variants differently and thus reject differences in survival and reproduction rates, score 0.

**Changes in Population**: To assess children’s ideas about change in population, they are asked to think about how the groups of variants in the selection scenario might have changed after some years have passed. They shall estimate if there might live more, less or the same number of variants with the disadvantageous (S3A) and advantageous trait (S3B; [89]).

In both cases, children receive a 0 if they assume that the population size would not have changed. If they assume that the population size would have changed but due to factors other than death (in S3A) or successful survival and reproduction (in S3B), they are scored 1. Conversely, when children assume that the population size would decrease due to death (S3A) or increase due to reproduction (S3B), they receive a score of 2.

**Speciation: Origin and extinction of species on Earth**: The belief of children about the origin of species is a well-studied topic (e.g., [87,91,93–96]). Following these studies, S4A ask the children whether they believe that the example species have lived on Earth forever, and if not, how did it come to live on Earth now. This is followed by the question of whether the children assume the example species would exist forever (S4B).

Children that assume that the example species has lived or will live on Earth forever are scored with 0. The intermediate category (1) is met when children assume the example species has not and will not live on Earth forever but cannot provide an accurate explanation (e.g., species got invented). More advanced explanations (2) acknowledge that the species has not lived forever but developed or evolved from another species, and will not live forever but will eventually die out or evolve into a different species. It is not necessary for the children to fully understand or explain evolutionary theory to meet the more accurate explanation criteria, as considering the age, the notion of evolution or development alone can be considered a first relatively advanced idea.

**Speciation: Common Ancestry**: The concept of common ancestry is captured here by two questions about the origin of the resemblance of closely related species (S4C) and their ability to procreate (S4D). When considered together, these two questions should indicate whether children have an idea of evolutionary relatedness that differs from their comprehension of familial ties. The children are presented with three closely related species (i.e., hooded crow, raven, and carrion crow in the animal condition and eagle fern, woodfern, and royal fern in the plant condition).

If the children provide an unsuitable answer or fail to give a specific reason (e.g., give a simple description; see [67]), they score 0. If they mention that the species belong to the same taxonomic class or family, they receive a score of 1. This is because taxonomic groups are composed of closely related species that share characteristics due to a common ancestor. While we do not expect children to understand the full implications of this statement, we consider this idea to be a first step toward an accurate explanation. If children suggest that the species are related (e.g., through evolution), they score 2. Again, they do not need to fully understand what evolution or relatedness is to meet the more advanced explanation’s criterion since it is considered a preliminary idea for their age. Similarly, in the second question, children score 0 if they assume the possibility of reproduction, 1 if they presume that reproduction is impossible without giving an adequate explanation, or 2 if they refer to the fact that the individuals belong to different species and thus cannot mate.

### Testing the CACIE in the field

We tested the CACIE in two steps. In a first phase, we conducted cross-sectional interviews in the United States to evaluate the digital implementation and the immediate coding through the integrated category system. To increase the likelihood of encountering all levels of responses without prior training, and to assess age related differences, we recruited a sample of younger children (aged 5–6 years, likely preliterate) as well as a sample of older children (aged 7–8 years, literate). In a second phase, we conducted cross-sectional interviews in Germany to evaluate the comparability of the translated version of the instrument. Additionally, we implemented a test-retest design to measure if a pre-post design would result in a testing effect.

The study was approved by the Human Subjects Research Review Committee of the Occidental College (USA; File N° FA22−22SHT) and by the Ethics Commission of the IPN Kiel (Germany, File N° 2023_02_AD).

#### First field testing of CACIE.

From winter 2022 until spring 2023 (October 23, 2022, to April 1, 2023), we used CACIE to conduct cross-sectional interviews with English-speaking children aged 5–6 and 7–8 years in the United States. They were recruited from local parks and tested on-site. Both caregivers and children were informed about the interview procedure, the aim of our study, the handling of their data, the meaning of consent, and their right to withdraw from the study at any time without negative consequences. Those families willing to participate signed a consent form and received a copy to take home. Additionally, the child’s consent was repeatedly obtained verbally during the interview (i.e., the child was asked if they wanted to continue with the interview), which was witnessed by the interviewer, the interrater, and the caregivers.

Children were randomly assigned to one of the two plant species (dandelion or apple tree) and one of the two animal species (red fox or brown-lipped snail) for the items V1, V2, V3, I1, I2, S1, S4A and S4B. Additionally, they were assigned to either a plant or an animal selection scenario for the items S2, S3, S4C, and S4D. One interviewer surveyed the children and rated their responses. They were accompanied by one to two interraters, who rated the children’s responses independently. In total, four different raters (including one of the authors) were involved in the data collection. Interrater training took place during piloting. The interrater reliability (IRR) was calculated with the Krippendorff’s alpha which is a common measure for categorical coding with more than two raters [55,111].

#### Second field testing of CACIE.

In spring 2023, we tested the CACIE in cross-sectional interviews with kindergarten children aged 5–6 in Germany. Additionally, we assessed a small sample in a test-retest design to investigate whether their performance would improve on the CACIE across multiple exposures without instruction. They were assessed twice and received a neutral reading intervention in between (i.e., evolutionary principles were not targeted in the children’s book). The participants were recruited in collaboration with local kindergartens (January 28, 2023 to February 28, 2023) and were tested on-site (February 20, 2023 to Mai 31, 2023). A consent form was distributed to the parents along with an information letter detailing the study, the procedure, data handling, and the meaning of consent. We also asked the parents to inform their children and seek their willingness to participate before signing the consent form. Immediately before the interviews, the children were again informed about the interview procedure, the aim of our study, the meaning of consent, and their right to withdraw at any time without negative consequences. Their consent was witnessed by the researcher, the interrater, and the kindergarten educator, and was again repeatedly obtained verbally during the interviews.

Concerning the children that were tested twice, the first test and the intervention took place on different days within one week, whereas the second test occurred one week after the intervention. The conditions were set to the red fox, the apple tree and the hooded crow selection scenario. The storybook reading was conducted in groups of two to three children. We chose the children’s book “The boy who grew a forest” [112] because it covers topics (i.e., forest ecosystem and environmental conservation) that are not assessed by the CACIE. The reading, including the questions asked and words explained by the reader, was scripted and practiced beforehand. The test-retest reliability (TRR) was assessed with the Intraclass Correlation Coefficient (ICC), which is a common measure for test-retest reliability suitable for small samples [113]. Two interraters were involved in the data collection, while the interviewer and reader remained consistent throughout the study. In between the field testings, we were able to improve our interrater training by using memory protocols of the U.S. sample that all raters rated and discussed beforehand (The training is available in the Supporting Information S2 File). IRR was again calculated with the Krippendorff’s alpha.

### Psychometric validation for the CACIE

To validate the responses generated by the CACIE, we followed the guidelines proposed by the American Educational Research Association [51], which recommends including evidence from (1) internal structure (i.e., alignment of the assessment’s structure and scoring with the theoretical constructs being measured), (2) relations to other variables (i.e., assessment of how test results relate to other variables in theoretically expected ways, such as age-related trends), (3) test content (i.e., evaluation of whether the items adequately and representatively capture the intended domain or construct), (4) consequences of testing (i.e., consideration of intended and unintended outcomes of using the test, including educational or developmental impacts), and (5) response processes (i.e., examination of the thought processes, interpretations, or strategies used by respondents when answering items to ensure they align with the intended construct). Those guidelines are commonly used in early childhood research and (evolution) education research to evaluate concept inventories as well as acceptance instruments (see [47,52,114]).

Evidence for internal structure will be provided by IRR and TRR. Evidence from relations to other variables will be generated using the demographic information of age, gender, and nationality. Given that, according to the NGSS Lead States [115], the topics of inheritance and variation of traits should be taught in first grade in the U.S., we expected age to correlate positively with the mean score of the items. Given that our data violated the assumptions required for parametric regression, we conducted a one-sided Jonckheere-Terpstra test with the alternative hypothesis set to *increasing*, to test this directional hypothesis. To explore potential gender differences, we conducted Mann-Whitney U-tests (also known as Wilcoxon rank-sum tests) on the five- and six-year-olds (*n* = 66) for each CACIE item as well as aggregated scores of the principles (variation, inheritance, selection) and a total mean of all items. We did not expect significant differences between genders as standardized science assessments for children that tested for gender differences (e.g., *Centre-of-Mass Test*, *Science-K Inventory*) found no significant differences between male and female participants [45,116,117]. However, it should be noted that most validation studies we are aware of did not examine the influence of gender. In addition, by comparing the two samples of five- and six-year-olds from the United States (*n* = 15, first field testing) and Germany (*n* = 51, second field testing), we examined potential differences between the nationalities again implementing Mann-Whitney U-tests for each CACIE item and the aggregated scores. Since variation, inheritance, and selection are not topics in kindergarten education in either of these countries, we do not expect significant differences between nationality and the mean score. To compare scores between animal and plant examples, we conducted a Wilcoxon signed-ranks test.

### Data processing and statistical implementation

The raw data were collected and organized in Microsoft® Excel® for Microsoft 365 (Version 2502), where initial data cleaning (e.g., removal of obvious entry errors, handling of missing codes, and variable labeling) was performed. Excel was also used for providing the descriptive statistics (i.e., means, standard deviations, minimum, and maximum values) and creating diagrams. Further data preparation, including filtering for specific subgroups and computing scale scores for each principle (i.e., variation, inheritance, and selection), as well as an overall score, was conducted in RStudio (Version 2025.05.1 + 513) using R (version 4.4.1). Data preparation relied on the *dplyr* and *tidyr* packages. Non-parametric analyses were performed using base R functions, including wilcox.test() for the Mann–Whitney U tests and the Wilcoxon signed-ranks tests (for comparing paired scores, i.e., between plant and animal examples), as well as the jonckheere.test() function from the *clinfun* package [118] for the Jonckheere-Terpstra trend tests. The ICCs were calculated using the ICC() function from the *psych* package. To visualize changes of the participants’ response scores in the test-retest design, a Sankey diagram was created using the *networkD3* package [119].

## Results

### First field testing of the CACIE

In total, 37 children participated in the cross-sectional interviews in the United States. Three of these children were part of the piloting. The main data collection comprised 15 children aged 5–6 years (*M* = 5.53, *SD* = 0.51, female: *n* = 13) and 19 children aged 7–8 years (*M* = 7.47, *SD* = 0.51, female: *n* = 10). The interviews took on average 18 minutes (*min* = 12; *max* = 28). The IRR yielded a total score of α = 0.84 (min = 0.61; max = 1), indicating an acceptable level of agreement between the raters [55] for all but one of the items that scored just below the generally accepted threshold of 0.67 (V2B: α = .61).

Overall, the children’s answers were distributed across all levels for all items. The items where the children scored highest were V3B (Effect of beneficial traits on longevity), I1A (Biological parents/ Sexual reproduction), and S1B (Different distribution of resources between members of a species; see Table 5). V1B (Variation in “inner” non-visible traits) and V2A (Between-parent variation) received the lowest scores. For most items, the scores for both age groups (five- to six-year-olds and seven- to eight-year-olds) spanned the full possible range from 0 to 2. However, there was one item where the five- to six-year-olds did not score higher than a 1 (i.e., V2A: Variation between parents). In contrast, the older children scored between 0 and 2 on all items.

**Table 5:** Descriptive statistics for all items by field testing and age group.

Concept	Subconcept	Item	First field testing				Second field testing^a^	
			*Country 1*				*Country 2*	
			ages 5–6 (*n* = 15)		ages 7–8 (*n* = 19)		ages 5–6 (*n* = 51)	
			*M* ± *SD*	range	*M* ± *SD*	range	*M* ± *SD*	range
**VARIATION**								
Individual Variation	Variation in heritable traits	V1A	0.55 ± 0.74	0–2	0.97 ± 0.80	0–2	0.76 ± 0.67	0–2
	Variation in “inner” non-visible traits	V1B	0.31 ± 0.60	0–2	0.61 ± 0.75	0–2	0.52 ± 0.63	0–2
Origin of Variation	Between-parent variation	V2A	0.29 ± 0.46	0–1	0.92 ± 0.6	0–2	0.23 ± 0.43	0–1
	Within-parent variation	V2B	0.95 ± 0.49	0–2	1.19 ± 0.65	0–2	0.73 ± 0.45	0–1
Differences in Fitness	Variation in beneficial traits	V3A	0.9 ± 0.72	0–2	1.05 ± 0.73	0–2	0.69 ± 0.55	0–2
	Effect of beneficial traits on longevity	V3B	1.54 ± 0.79	0–2	1.86 ± 0.42	0–2	1.59 ± 0.68	0–2
**INHERITANCE**								
Reproduction	Biological parents/ Sexual reproduction	I1A	1.56 ± 0.70	0–2	1.5 ± 0.74	0–2	1.33 ± 0.87	0–2
	Hyperfecundity and population size	I1B	0.93 ± 0.75	0–2	1.47 ± 0.56	0–2	1.05 ± 0.54	0–2
Inheritance of Variation	Resemblance in families	I2A	0.41 ± 0.67	0–2	0.71 ± 0.78	0–2	1.01 ± 0.97	0–2
	Variation between siblings	I2B	0.78 ± 0.52	0–2	1.1 ± 0.72	0–2	0.72 ± 0.45	0–1
**SELECTION**								
Limited Resources	Limited resources in the environment	S1A	0.75 ± 0.89	0–2	1 ± 0.97	0–2	0.71 ± 0.88	0–2
	Different distribution of resources between members of a species	S1B	1.36 ± 0.68	0–2	1.66 ± 0.63	0–2	1.12 ± 0.83	0–2
Differences in Reproduction and Survival Rate	Different survival rates within a population due to different traits	S2A	0.86 ± 0.95	0–2	1.26 ± 0.93	0–2	0.79 ± 0.91	0–2
	Different reproduction rates within a population due to different traits	S2B	1 ± 0.96	0–2	1.32 ± 0.89	0–2	0.66 ± 0.88	0–2
Changes in Population	Change in trait frequency after disadvantage	S3A	0.92 ± 0.95	0–2	1.47 ± 0.84	0–2	1.11 ± 0.91	0–2
	Change in trait frequency after advantage	S3B	0.62 ± 0.87	0–2	1.06 ± 0.87	0–2	0.76 ± 0.79	0–2
Origin and extinction	Origin of species	S4A	0.69 ± 0.66	0–2	1.11 ± 0.7	0–2	0.80 ± 0.57	0–2
	Extinction of species	S4B	0.55 ± 0.87	0–2	0.81 ± 0.94	0–2	0.71 ± 0.81	0–2
Common Ancestry	Families and phylogeny	S4C	0.46 ± 0.66	0–2	0.67 ± 0.59	0–2	0.50 ± 0.74	0–2
	Species boundaries	S4D	0.54 ± 0.78	0–2	0.78 ± 0.94	0–2	0.69 ± 0.76	0–2

^a^These results include only the pre-test and not the retest values.

Overall, older children outperformed younger ones on all items except I1A, where younger children scored slightly higher (0.06), though the difference was not significant. Overall, age seems to be positively correlated with mean scores as the Jonckheere-Terpstra tests revealed significant age-related differences in the aggregated scores for items related to variation (*p* < .001), inheritance (*p* = .001), and selection (*p* < .001; see Table 6). These differences in mean values can be attributed to statistically significant differences in the items V2A, V2B, V3A, V3B, I1B, I2B, S1B, S2A, S2B, S3A, and S4A.

**Table 6:** Overview of statistical test results on CACIE scores by demographic variables.

Concept	Subconcept	Variable	Mann-Whitney U			Mann-Whitney U			Jonckheere-Terpstra		
			(gender)			(nationality)			(age)		
			*5- and 6 year olds (n = 66)*			*5- and 6 year olds (n = 66)*			*5 to 8 year olds (n = 85)*		
			W	p	Sign.	W	p	Sign.	JT	p	Sign.
**OVERALL**			2197.5	.501		1391.0	.627		6307.5	.000	***
**VARIATION**			2430.0	.076		1387.0	.610		5978.5	.000	***
Individual Variation	Variation in heritable traits	V1A	2127.5	.467		1184.5	.102		4788.0	.122	
	Variation in “inner” non-visible traits	V1B	2113.5	.086		1100.5	.071		4389.0	.250	
Origin of Variation	Between-parent variation	V2A	603.5	.180		546.5	.603		2533.0	.000	***
	Within-parent variation	V2B	966.0	.400		831.5	.069		2866.0	.000	***
Differences in Fitness	Variation in beneficial traits	V3A	2171.5	.104		1612.0	.174		5054.0	.013	*
	Effect of beneficial traits on longevity	V3B	1474.5	.830		1153.5	.946		4259.0	.010	*
**INHERITANCE**			1846.0	.449		1281.0	.335		5513.5	.001	***
Reproduction	Biological parents/ Sexual reproduction	I1A	1949.5	.769		1479.0	.284		4563.5	.085	
	Hyperfecundity and population size	I1B	1748.0	.805		1216.5	.350		5242.0	.000	***
Inheritance of Variation	Resemblance in families	I2A	879.5	.162		519.5	.011	*	2366.0	.572	
	Variation between siblings	I2B	842.0	.603		698.0	.683		2326.5	.021	*
**SELECTION**			2113.0	.687		1476.5	.949		5782.0	.000	***
Limited Resources	Limited resources in the environment	S1A	1935.5	.836		1393.0	.819		4685.5	.073	
	Different distribution of resources between members of a species	S1B	1753.5	.955		1494.5	.207		5185.5	.000	***
Differences in Reproduction and Survival Rate	Different survival rates within a population due to different traits	S2A	407.0	.908		312.5	.821		1188.5	.010	**
	Different reproduction rates within a population due to different traits	S2B	378.5	.358		318.0	.234		1105.0	.001	**
Changes in Population	Change in trait frequency after disadvantage	S3A	535.5	.112		273.0	.533		1238.0	.020	*
	Change in trait frequency after advantage	S3B	472.0	.438		264.0	.493		1132.5	.050	
Origin and extinction	Origin of species	S4A	1612.5	.854		1122.5	.308		4588.0	.007	**
	Extinction of species	S4B	1794.0	.914		1163.0	.249		4083.0	.403	
Common Ancestry	Families and phylogeny	S4C	132.0	.569		142.5	1.000		569.5	.071	
	Species boundaries	S4D	376.5	.656		258.5	.492		1002.0	.280	

*** p < 0.001, ** p < 0.01, * p < 0.05.

**Fig 2:**
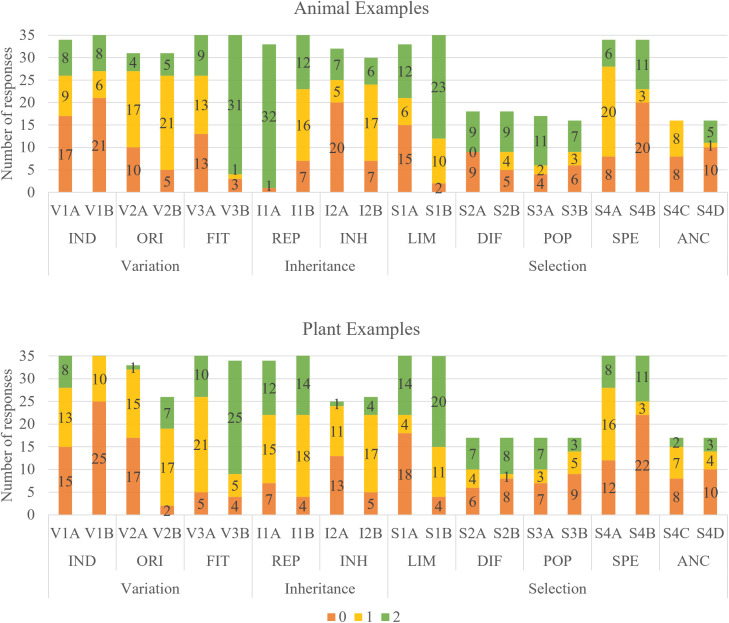
Number of answers that were rated unscientific (0), intermediate (1), or more accurate (2) for the ten key concepts for animal and plant examples (*n* = 34).

The answers showed to be mostly consistent across the animal and plant conditions (see Fig 2). Only two items differed between the conditions: The children scored significantly higher for V2A (**p* *< .05) and I1A (*p* < .001) in the animal condition. Comparisons between the species showed no significant differences in any of the items, either between the plant species (dandelion vs. apple tree) or the animal species (red fox vs. brown-lipped snail).

### Second field testing of the CACIE

In total, cross-sectional data was collected of 51 children (age: *M* = 5.71 years; *SD *= 0.46) in Germany*.* In terms of gender, 27 of the 51 children were female. Fourteen of those children (female: **n* *= 9; age: *M* = 5.4 years; *SD* = .51) also participated in the neutral intervention and a retest. The interviews took on average 12 minutes (min = 8; max = 17). The IRR scored a Krippendorff’s alpha of α = 0.95 with all items being above the acceptable threshold of 0.67 (min = 0.71; max = 1), indicating an acceptable level of agreement between the raters.

### Cross-sectional design

The items where the children scored highest were again V3B (Effect of beneficial traits on longevity), I1A (Biological parents/ Sexual reproduction), and S1B (Different distribution of resources between members of a species; see Table 5). V2A (Variation in “inner” non-visible traits) and V1B (Variation in “inner” non-visible traits) again received the lowest score. The Mann-Whitney U-tests did not reveal any gender differences for any of the items (see Table 6). A comparison between the 5- and 6-year-olds of the two countries showed that the children from Germany scored significantly higher on the items I2A (on average 0.60 points higher, p < .05; see Tables 5 and 6). However, these differences did not manifest themselves in the inheritance score or any of the other aggregated scores.

#### Test-retest design.

**Fig 3:**
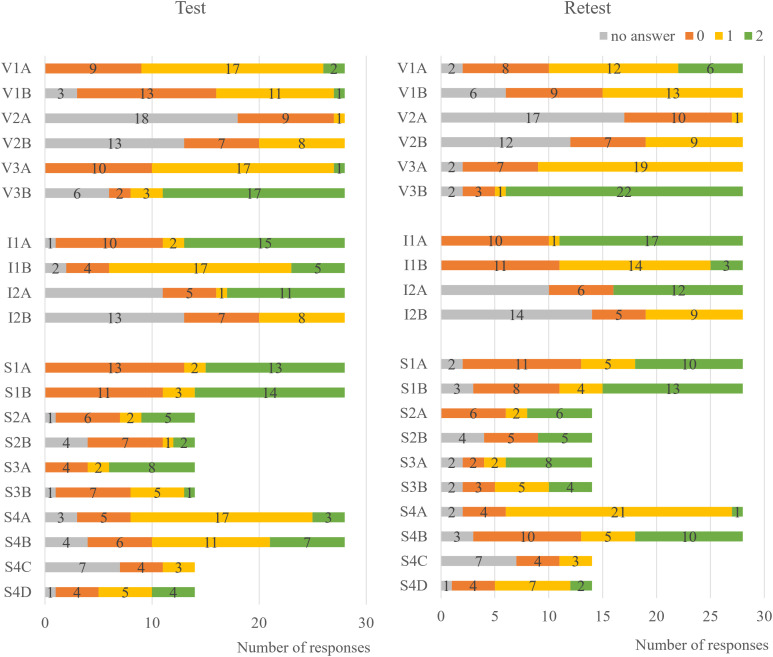
Number of answers that were rated unscientific (0), intermediate (1), or more accurate (2) for the ten key concepts* (*n* = 14). V1 Individual variation, V2 Origin of variation, V3 Differential Fitness, I1 Reproduction, I2 Inherited variation, S1 Limited resources, S2 Differential survival & reproduction rate, S3 Change in population, S4 Speciation.

Most responses that the children gave were consistent throughout both testings (*n* = 277 responses; see Fig 3). TRR was found to be moderately stable with an ICC of.68 [113]. Three items (V2B, V3B, S4B) fell below the acceptable threshold of.50.

For the responses that differed between the first and second interview, there was no systematic but an equal flow to lower (*n* = 75 responses) or higher scores (*n* = 87 responses; see Flow Chart in the Supporting Information S3 Fig).

### Psychometric validation for the CACIE

Below we summarize how the CACIE performed based on the different validity evidences (Table 7).

**Table 7:** Validity evidence of the CACIE (structured by the AERA guidelines).

Validity evidence	Component		Validity argument
Internal structure	Reliability	IRR	Acceptable level of agreement between the raters (see *First/ Second field testing of the CACIE*)
		TRR	Moderately stable test-retest reliability (see *Intraclass correlation coefficient*)
Relations to other variables	Demographics		1. Significant positive correlation between age and mean score 2. No significant difference between gender and scores (see *First/ Second field testing of the CACIE*)
	Nationality		No significant difference between nationality and mean score for (except for I2A; see *Second field testing of the CACIE*)
Test content	Theoretical description		An empirically validated and well-established theoretical framework was used to guide item selection and creation, ensuring relevance and alignment with the construct being measured. Additionally, published instruments and interviews were reviewed to inform the development of new items or adapt pre-existing ones (see *Format and Item Design*).
	Pilot test		Empirical evidence was gathered in four pilotings to identify flaws or limitations, and to make adjustments to improve content validity (see *Development of the CACIE*).
Response processes	Observation		Evidence from the observations during the first data collection was used to revise and improve the CACIE [54]
Consequences of testing			Potential negative consequences of taking the test were carefully considered during its development process and is discussed in S4 File. We received exclusively positive feedback from the children and teachers.

## Discussion

In this article, we introduce the Conceptual Assessment of Children’s Ideas about Evolution (CACIE; guidelines for using the CACIE are available as Supporting Information S4 File), comprising a set of 20 interview-based items (including stem and follow-up questions, visual prompts, and a three scaled category system) about the evolutionary principles variation, inheritance, and selection, and reported its development as well as its validation based on the AERA Standards for Educational and Psychological Testing [51]. Standardized instruments to measure young children’s ideas about evolution are lacking, and many studies with pre-literate children have small sample sizes (see [38]) due to time and effort associated with transcription as well as ethical and privacy issues associated with audio recording minors [104]. We developed the CACIE as an attempt to resolve these issues. The first version took an average of 34 minutes to complete and required the interviewer to manage printed illustrations, as well as audio-record and transcribe the children’s responses [54]. The latest version can be handled more easily through digital implementation and takes half the time, making it possible to conduct the interview without breaks and increasing the likelihood of sustained engagement throughout the assessment. Also, the training with authentic children’s responses increased the reliability of the interrating (see Supporting Information S2 File).

The development of the CACIE was guided by a strong and well-established theoretical framework, ensuring the relevance and alignment of the construct being measured. We extensively reviewed published instruments and conducted interviews to inform the creation of new items or adapt pre-existing ones. The CACIE underwent four pilot tests to gather information about how children respond to the questions and illustrations, allowing for its refinement and enhancement. Our findings suggest that the CACIE is unlikely to exhibit floor or ceiling effects (i.e., large number of participants scoring at the lowest or highest possible value on a measure, respectively, limiting the ability to detect variation in the sample or changes over time, such as from pre- to post-test) when utilized in an intervention study, and that the categories (i.e., the scoring rules) seem to align with age-appropriate definitions of the key concepts. This conclusion is supported by the fact that, even though the older children (aged 7–8 years) tended to perform significantly better, the majority of children sampled had scores ranges including the highest score regardless of age or nationality. The younger children (5–6 years) only scored between 0 and 1 on V2A (between-parent variation). Additionally, for the items V2B (within-parent variation) and I2B (variation between siblings), the children from Germany also only scored between 0 and 1, whereas one six-year-old from the U.S. sample achieved scoring a 2. These three items have in common that they require the children to reason about variation between or within families and to infer parental traits from offspring characteristics. We anticipated that understanding the origin of variation would be particularly challenging for the young target group of this study, as it involves reasoning about genetics (e.g., mutations, recombination; [65]). By focusing on parental variation, we aimed to make the concept more tangible by shifting the subject from a molecular to an individual scale (see also [67]). However, previous research examining reasoning about variation and inheritance at the individual scale has shown that young children do recognize these concepts within families but, particularly at younger ages, exhibit a sex-matching bias (i.e., expecting offspring to resemble their same-sex parent; [77]). Thus, achieving a score of 2 on these concepts may still be more challenging than on the others. Future research could explore whether simpler questions could be designed for these concepts (e.g., framing the inherited variation task in a top-down manner (parents to offspring)) or whether five- to six-year-olds children might be able to achieve the highest score (i.e., a [2]) after an intervention. Notably, the children we interviewed had no specialized background, suggesting that prior training might be necessary for young children to succeed on these items. However, it should be noted that with larger sample sizes, younger children may also reach the highest scores, although likely at a lower frequency than older children.

As expected, the statistical analyses revealed that children performed better with age. Older children may have better understood the questions or the underlying topics of variation and inheritance, which are traditionally introduced in the first grade [115]. Additionally, the general lack of significant differences between nationality and gender in all but three items suggests that the CACIE has no obvious bias towards specific demographic groups.

Our findings also provide evidence that the CACIE is psychometrically sound. For instance, IRR and TRR analyses indicate that the category system of the CACIE leads to consistent outcomes among raters, especially in combination with a prior interrater training that uses realistic training data, and reasonable stability and consistency in the measures over time. While the majority of items on the CACIE met the ICC threshold, there were three items that did not. Therefore, caution should be exercised when using these particular items in studies that employ the CACIE. However, it is worth noting that young children are known to provide less reliable answers compared to their older counterparts [120]. Importantly, our results showed that the items did not lead to consistently higher answers, which indicates that a potential learning effect can be ruled out.

## Limitations and future studies

The CACIE is a novel tool designed to evaluate children’s ideas about the evolutionary principles of variation, inheritance, and selection. Given its exploratory state there are still several limitations that would need to be addressed in future studies. So far, the CACIE does not provide a normative database that would allow to compare an individual’s performance against a normed group and is not suited for use by teachers. Instead, the CACIE may prove useful to researcher in the field to assess educational material or interventions related to evolution, such as the multitude of children’s books published about the topic of evolution (see [37]). Consequently, the subsequent step should be to test the CACIE in combination with interventions that might influence children’s ideas about the evolutionary principles (e.g., the children’s books interventions of [121,122]; see also [85,88,92]). Thus, we would like to see the CACIE being tested with greater samples and additional variables (e.g., social status, religion) as well as in different settings to gather more evidence of its validity or to get insights into how it could be further improved. In this regard, it would be valuable to compare the CACIE’s effectiveness to other standardized tools for evaluating kindergarten children’s school readiness or conceptual scientific knowledge (e.g., [39]) or to future standardized tools designed to measure the same construct (i.e., ideas about evolutionary concepts). Following studies could also translate the CACIE into other languages to broaden the target group and enable comparison between different cultural and social contexts.

Another area of improvement concerns the scoring of the CACIE, which involves a degree of interpretive judgment. First, the formal distinctions between levels of evolutionary thinking (e.g., ambiguous vs. advanced ideas) are, to some extent, arbitrary. Second, interpretive decisions were necessary during scoring, as kindergarten children often hold complex and nuanced ideas but may not always be able to articulate them fully due to their developing language skills (see also [104]). By grounding the distinctions in prior empirical studies and clearly articulating the rationale for each category, we aimed to capture and transparently communicate meaningful differences in children’s ideas about evolution. Nevertheless, these distinctions should be further tested and refined in future research.

The subconcepts we have addressed, and consequently, the items we have included, were selected based on informed choices, as outlined in the methods section. However, it is important to acknowledge that there may be additional relevant items that researchers could propose. Notably, recent research by Sá-Pinto et al. [36] highlights the positive impact of incorporating activities that model biological evolution with a focus on sexual selection, enhancing the understanding of evolutionary processes among third and fourth-grade students. While the current version of the CACIE does not encompass sexual selection, it would be valuable to explore the possibility of integrating this concept in future iterations. This should of course be done only after careful consultation with sensitivity readers and child psychologists, to ensure that the questions are age-appropriate and avoid promoting gendered behavioral expectations or creating social stress in the children being interviewed. So far, the assessment has only been administered to children aged 5–6 and 7–8, who have yet to receive formal instruction in evolution. Future research could validate the results by including it in a teaching unit or testing older participants with prior education in evolution. Finally, deciding between quantitative and qualitative data collection is always a trade-off, and the quantitative output of the CACIE will not capture the full complexity of children’s ideas. To mitigate this limitation, researchers can still supplement the CACIE data with audio recordings of the interviews. This would allow for a more nuanced examination of children’s ideas and provide additional qualitative insights. Researchers are encouraged to explore ways to improve the CACIE, whether through modifications, extensions, or new versions that address the current limitations and enhance its overall effectiveness.

## Conclusion

In life sciences education research, concept inventories and standardized assessments help explore learners’ ideas and inform the design of interventions and curricula. Given the limited time devoted to science education in kindergarten (see [123]), it is even more important to carefully assess learning opportunities [124]. Despite the widespread use of such tools in life sciences education, there is still a noticeable lack of assessment tools specifically designed to evaluate the ideas of young learners (see [46]), which could be used in the design and evaluation of educational material. The limitations posed by limited sample sizes in studies examining this age group often stem from issues concerning transcription, ethical considerations, and privacy issues associated with audio recordings of minors [104]. Additionally, assessments in early childhood research have been criticized for not following the AERA guidelines on validation practices (see [52]). The development of standardized tools has the potential to significantly improve research methodologies and allow for more comparable evaluations of learning materials and opportunities. By providing a tool designed to assess the evolution-related ideas of young, preliterate children, the introduction of the CACIE is a seminal effort in this regard. It includes a wide range of key concepts related to evolution as well as a variety of examples from the animal and plant kingdoms, making it adaptable to specific research objectives. The digital implementation of this tool makes it easy to use and protects the participants’ privacy by avoiding collecting sensitive data. So far, it cannot serve the purpose as a norm-referenced assessment but could contribute to the evaluation of educational material.

## Supporting information

S1 File CACIE items and categories (in the order of the interview). (DOCX)

S2 File Training material. (XLSX)

S3 Fig Flowchart of children that scored lower, higher, or the same on the items in the retesting (n = 14). (PNG)

S4 File Guidelines for using the CACIE. (DOCX)

## References

[1] Heams T, Huneman P, Lecointre G, Silberstein M. Handbook of evolutionary thinking in the sciences. Dordrecht: Springer Netherlands; 2015.

[2] Herron JC, Freeman F. Evolutionary analysis. 5th ed. Boston: Pearson; 2014.

[3] Gould SJ. The structure of evolutionary theory. Cambridge, Mass: Belknap Press of Harvard University Press; 2002.

[4] Mayr E. What evolution is. New York: Basic Books; 2001.

[5] Coley JD, Tanner KD. Common origins of diverse misconceptions: cognitive principles and the development of biology thinking. CBE Life Sci Educ. 2012;11(3):209–15. doi: 10.1187/cbe.12-06-0074 22949417 PMC3433289

[6] Gregory TR. Understanding Natural Selection: Essential Concepts and Common Misconceptions. Evo Edu Outreach. 2009;2(2):156–75. doi: 10.1007/s12052-009-0128-1

[7] Harms U, Reiss MJ. The present status of evolution education. In: Harms U, Reiss MJ, editors. Evolution education re-considered: Understanding what works. Cham, Switzerland: Springer; 2019. p. 1–19.

[8] Richard M, Coley JD, Tanner KD. Investigating Undergraduate Students’ Use of Intuitive Reasoning and Evolutionary Knowledge in Explanations of Antibiotic Resistance. CBE Life Sci Educ. 2017;16(3):ar55. doi: 10.1187/cbe.16-11-0317 28821540 PMC5589435

[9] Campos R, Vieira de Almeida Menezes M d. C, Alves de Sousa R. Identifying alternative conceptions about evolution in Portuguese high-school students: A reflection based on new and published data. X Congreso Internacional sobre Investicación en Didáctica de las Ciencias. 2017.

[10] Champagne Queloz A, Klymkowsky MW, Stern E, Hafen E, Köhler K. Diagnostic of students’ misconceptions using the Biological Concepts Instrument (BCI): A method for conducting an educational needs assessment. PLoS One. 2017;12(5):e0176906. doi: 10.1371/journal.pone.0176906 28493960 PMC5426623

[11] Karataş A. Preservice Science Teachers’ Misconceptions About Evolution. JETS. 2020;8(2):38. doi: 10.11114/jets.v8i2.4690

[12] Rice JW, Clough MP, Olson JK, Adams DC, Colbert JT. University faculty and their knowledge & acceptance of biological evolution. Evo Edu Outreach. 2015;8(1). doi: 10.1186/s12052-015-0036-5

[13] Barnes ME, Evans EM, Hazel A, Brownell SE, Nesse RM. Teleological reasoning, not acceptance of evolution, impacts students’ ability to learn natural selection. Evo Edu Outreach. 2017;10(1). doi: 10.1186/s12052-017-0070-6

[14] Schramm T, Schmiemann P. Teleological pitfalls in reading evolutionary trees and ways to avoid them. Evo Edu Outreach. 2019;12(1). doi: 10.1186/s12052-019-0112-3

[15] Shtulman A. Why people do not understand evolution: an analysis of the cognitive barriers to fully grasping the unity of life. Skeptic Magazine; 2011;16.

[16] Varella MAC. The Biology and Evolution of the Three Psychological Tendencies to Anthropomorphize Biology and Evolution. Front Psychol. 2018;9:1839. doi: 10.3389/fpsyg.2018.01839 30327628 PMC6174228

[17] Beggrow EP, Sbeglia GC. Do disciplinary contexts impact the learning of evolution? Assessing knowledge and misconceptions in anthropology and biology students. Evo Edu Outreach. 2019;12(1). doi: 10.1186/s12052-018-0094-6

[18] Fiedler D, Sbeglia GC, Nehm RH, Harms U. How strongly does statistical reasoning influence knowledge and acceptance of evolution? J Res Sci Teach. 2019;56(9):1183–206. doi: 10.1002/tea.21547

[19] Ha M, Nehm RH. Darwin’s Difficulties and Students’ Struggles with Trait Loss: Cognitive-Historical Parallelisms in Evolutionary Explanation. Sci & Educ. 2013;23(5):1051–74. doi: 10.1007/s11191-013-9626-1

[20] Nehm RH, Poole TM, Lyford ME, Hoskins SG, Carruth L, Ewers BE, et al. Does the Segregation of Evolution in Biology Textbooks and Introductory Courses Reinforce Students’ Faulty Mental Models of Biology and Evolution? Evo Edu Outreach. 2008;2(3):527–32. doi: 10.1007/s12052-008-0100-5

[21] Sanders M, Makotsa D. The possible influence of curriculum statements and textbooks on misconceptions: The case of evolution. Educ as Change. 2016. doi: 10.17159/1947-9417/2015/555

[22] Berkman MB, Sandell Pacheco J, Plutzer E. Evolution and creationism in America’s classrooms: A national portrait. PLoS Biology. 2008;6:920–4. doi: 10.1371/journal.pbio.0060124.g001 PMC2386841 18494560

[23] Siani M, Yarden A. “Evolution? I Don’t Believe in It”. Sci & Educ. 2020;29(2):411–41. doi: 10.1007/s11191-020-00109-7

[24] Aptyka H, Fiedler D, Großschedl J. Effects of situated learning and clarification of misconceptions on contextual reasoning about natural selection. Evo Edu Outreach. 2022;15(1). doi: 10.1186/s12052-022-00163-5

[25] Mead R, Hejmadi M, Hurst LD. Teaching genetics prior to teaching evolution improves evolution understanding but not acceptance. PLoS Biol. 2017;15(5):e2002255. doi: 10.1371/journal.pbio.2002255 28542179 PMC5441579

[26] Neubrand C, Harms U. Tackling the difficulties in learning evolution: effects of adaptive self-explanation prompts. Journal of Biological Education. 2016;51(4):336–48. doi: 10.1080/00219266.2016.1233129

[27] Steinwachs J, Martens H. Addressing student conceptions in evolution classes: professional vision practices of preservice and in-service biology teachers. Evo Edu Outreach. 2022;15(1). doi: 10.1186/s12052-022-00174-2

[28] Lee TW, Grogan KE, Liepkalns JS. Making evolution stick: using sticky notes to teach the mechanisms of evolutionary change. Evolution (N Y). 2017;10:11. doi: 10.1186/s12052-017-0074-2 36873251 PMC9979970

[29] Metz KE, Cardace A, Berson E, Ly U, Wong N, Sisk-Hilton S, et al. Primary Grade Children’s Capacity to Understand Microevolution: The Power of Leveraging Their Fruitful Intuitions and Engagement in Scientific Practices. Journal of the Learning Sciences. 2019;28(4–5):556–615. doi: 10.1080/10508406.2019.1667806

[30] Fischer J, Jansen T, Möller J, Harms U. Measuring biology trainee teachers’ professional knowledge about evolution—introducing the Student Inventory. Evo Edu Outreach. 2021;14(1). doi: 10.1186/s12052-021-00144-0

[31] Hartelt T, Martens H, Minkley N. Teachers’ ability to diagnose and deal with alternative student conceptions of evolution. Science Education. 2022;106(3):706–38. doi: 10.1002/sce.21705

[32] Brandt M, Groom Q, Magro A, Misevic D, Narraway CL, Bruckermann T, et al. Promoting scientific literacy in evolution through citizen science. Proc Biol Sci. 2022;289(1980):20221077. doi: 10.1098/rspb.2022.1077 35946159 PMC9363982

[33] Borgerding LA, Raven S. Children’s ideas about fossils and foundational concepts related to fossils. Science Education. 2017;102(2):414–39. doi: 10.1002/sce.21331

[34] Horwitz P, McIntyre CA, Lord TL, O’Dwyer LM, Staudt C. Teaching ‘Evolution readiness’ to fourth graders. Evo Edu Outreach. 2013;6(1). doi: 10.1186/1936-6434-6-21

[35] Kelemen D. The Magic of Mechanism: Explanation-Based Instruction on Counterintuitive Concepts in Early Childhood. Perspect Psychol Sci. 2019;14(4):510–22. doi: 10.1177/1745691619827011 31017833

[36] Sá-Pinto X, Pessoa P, Pinto A, Cardia P, Bernardino Lopes J. The Impact of Exploring Sexual Selection on Primary School Students’ Understanding of Evolution. CEPSj. 2023;13(1):121–41. doi: 10.26529/cepsj.1508

[37] Adler IK, Fiedler D, Harms U. Darwin’s tales: A content analysis of how evolution is presented in children’s books. PLoS One. 2022;17(7):e0269197. doi: 10.1371/journal.pone.0269197 35830379 PMC9278771

[38] Bruckermann T, Fiedler D, Harms U. Identifying precursory concepts in evolution during early childhood – a systematic literature review. Studies in Science Education. 2020;57(1):85–127. doi: 10.1080/03057267.2020.1792678

[39] Greenfield DB. Assessment in early childhood science education. In: Trundle KC, Saçkes M, editors. Research in early childhood science education. Dordrecht, Heidelberg, New York, London: Springer; 2015. p. 353–80. doi: 10.1007/978-94-017-9505-0

[40] Clarke-Midura J, Lee VR, Shumway JF, Silvis D, Kozlowski JS, Peterson R. Designing formative assessments of early childhood computational thinking. Early Childhood Research Quarterly. 2023;65:68–80. doi: 10.1016/j.ecresq.2023.05.013

[41] Gao X, Li P, Shen J, Sun H. Reviewing assessment of student learning in interdisciplinary STEM education. IJ STEM Ed. 2020;7(1). doi: 10.1186/s40594-020-00225-4

[42] Roach AT, McGrath D, Wixson C, Talapatra D. Aligning an Early Childhood Assessment to State Kindergarten Content Standards: Application of a Nationally Recognized Alignment Framework. Educational Measurement. 2010;29(1):25–37. doi: 10.1111/j.1745-3992.2009.00167.x

[43] Vitiello VE, Whittaker JV, Mulcahy C, Kinzie MB, Helferstay L. Reliability and Validity of the Preschool Science Observation Measure. Early Education and Development. 2018;30(2):196–215. doi: 10.1080/10409289.2018.1544814

[44] Samarapungavan A, Mantzicopoulos P, Patrick H, French B. The Development and Validation of the Science Learning Assessment (SLA): A Measure of Kindergarten Science Learning. Journal of Advanced Academics. 2009;20(3):502–35. doi: 10.1177/1932202x0902000306

[45] Koerber S, Osterhaus C. Individual Differences in Early Scientific Thinking: Assessment, Cognitive Influences, and Their Relevance for Science Learning. Journal of Cognition and Development. 2019;20(4):510–33. doi: 10.1080/15248372.2019.1620232

[46] Pedaste M, Baucal A, Reisenbuk E. Towards a science inquiry test in primary education: development of items and scales. IJ STEM Ed. 2021;8(1). doi: 10.1186/s40594-021-00278-z

[47] Furrow RE, Hsu JL. Concept inventories as a resource for teaching evolution. Evo Edu Outreach. 2019;12(1). doi: 10.1186/s12052-018-0092-8

[48] Anderson DL, Fisher KM, Norman GJ. Development and evaluation of the conceptual inventory of natural selection. J Res Sci Teach. 2002;39(10):952–78. doi: 10.1002/tea.10053

[49] Kalinowski ST, Leonard MJ, Taper ML. Development and Validation of the Conceptual Assessment of Natural Selection (CANS). CBE Life Sci Educ. 2016;15(4):ar64. doi: 10.1187/cbe.15-06-0134 27856552 PMC5132361

[50] Nehm RH, Beggrow EP, Opfer JE, Ha M. Reasoning About Natural Selection: Diagnosing Contextual Competency Using the ACORNS Instrument. The American Biology Teacher. 2012;74(2):92–8. doi: 10.1525/abt.2012.74.2.6

[51] American Educational Research Association, American Psychological Association, National Council on Measurement in Education. Standards for educational and psychological testing. Washington, DC: American Educational Research Association; 2014.

[52] Barghaus KM, Fantuzzo JW, Buek K, Gullo DF. Neglected validities: A diagnostic look at the state of early childhood assessment. Early Childhood Research Quarterly. 2022;58:287–99. doi: 10.1016/j.ecresq.2021.09.007

[53] Sá-Pinto X, Pinto A, Ribeiro J, Sarmento I, Pessoa P, Rodrigues LR, et al. Following Darwin’s footsteps: Evaluating the impact of an activity designed for elementary school students to link historically important evolution key concepts on their understanding of natural selection. Ecol Evol. 2021;11(18):12236–50. doi: 10.1002/ece3.7849 34594496 PMC8462140

[54] Adler IK, Fiedler D, Harms U. About birds and bees, snails and trees: Children's ideas on animal and plant evolution. Science Education. 2024;108(5):1356–1391. 10.1002/sce.21873

[55] Krippendorff K. Content analysis: An introduction to its methodology. 3rd ed. Los Angeles, London, New Delhi, Singapore: Sage; 2013.

[56] Ha M, Baldwin BC, Nehm RH. The Long-Term Impacts of Short-Term Professional Development: Science Teachers and Evolution. Evo Edu Outreach. 2015;8(1). doi: 10.1186/s12052-015-0040-9

[57] Bohlin G, Göransson A, Höst GE, Tibell LAE. A Conceptual Characterization of Online Videos Explaining Natural Selection. Sci & Educ. 2017;26(7–9):975–99. doi: 10.1007/s11191-017-9938-7

[58] Opfer JE, Nehm RH, Ha M. Cognitive foundations for science assessment design: Knowing what students know about evolution. J Res Sci Teach. 2012;49(6):744–77. doi: 10.1002/tea.21028

[59] Peel A, Zangori L, Friedrichsen P, Hayes E, Sadler T. Students’ model-based explanations about natural selection and antibiotic resistance through socio-scientific issues-based learning. International Journal of Science Education. 2019;41(4):510–32. doi: 10.1080/09500693.2018.1564084

[60] Tibell LAE, Harms U. Biological Principles and Threshold Concepts for Understanding Natural Selection. Sci & Educ. 2017;26(7–9):953–73. doi: 10.1007/s11191-017-9935-x

[61] Alred AR, Doherty JH, Hartley LM, Harris CB, Dauer JM. Exploring student ideas about biological variation. International Journal of Science Education. 2019;41(12):1682–700. doi: 10.1080/09500693.2019.1635289

[62] Batzli JM, Knight JK, Hartley LM, Maskiewicz AC, Desy EA. Crossing the Threshold: Bringing Biological Variation to the Foreground. CBE Life Sci Educ. 2016;15(4):es9. doi: 10.1187/cbe.15-10-0221 27856553 PMC5132383

[63] Shtulman A. Qualitative differences between naïve and scientific theories of evolution. Cogn Psychol. 2006;52(2):170–94. doi: 10.1016/j.cogpsych.2005.10.001 16337619

[64] Mayr E. The growth of biological thought: diversity, evolution, and inheritance. 2nd ed. Cambridge, Mass.: Harvard Univ. Pr; 1982.

[65] Kirschner M, Gerhart J. The plausibility of life: Resolving Darwin’s dilemma. New Haven: Yale University Press; 2005.

[66] Kimura M. The neutral theory of molecular evolution and the world view of the neutralists. Genome. 1989;31(1):24–31. doi: 10.1139/g89-009 2687096

[67] Gormley K, Birdsall S, France B. Same, same but different! Exploring children’s understandings of within-species variation. Journal of Biological Education. 2022;58(3):530–51. doi: 10.1080/00219266.2022.2081244

[68] Shtulman A, Schulz L. The relation between essentialist beliefs and evolutionary reasoning. Cogn Sci. 2008;32(6):1049–62. doi: 10.1080/03640210801897864 21585442

[69] Emmons NA, Kelemen DA. Young children’s acceptance of within-species variation: Implications for essentialism and teaching evolution. J Exp Child Psychol. 2015;139:148–60. doi: 10.1016/j.jecp.2015.05.011 26101878

[70] Ibourk A, Williams M, Opperman A, Cisterna D, Nazar CR, Xie Y. Young students’ understanding of the relationship between inheritance and variation of traits using structural equation modeling. Science Education. 2018;102(6):1201–38. doi: 10.1002/sce.21470

[71] Carey S. Conceptual change in childhood. Cambridge, Mass.: MIT Pr; 1985.

[72] Ergazaki M, Alexaki A, Papadopoulou C, Kalpakiori M. Young Children’s Reasoning About Physical & Behavioural Family Resemblance: Is There a Place for a Precursor Model of Inheritance? Sci & Educ. 2013;23(2):303–23. doi: 10.1007/s11191-013-9594-5

[73] Waxman S, Medin D, Ross N. Folkbiological reasoning from a cross-cultural developmental perspective: early essentialist notions are shaped by cultural beliefs. Dev Psychol. 2007;43(2):294–308. doi: 10.1037/0012-1649.43.2.294 17352540

[74] Williams JM, Smith LA. Concepts of kinship relations and inheritance in childhood and adolescence. Br J Dev Psychol. 2010;28(Pt 3):523–46. doi: 10.1348/026151009x449568 20849032

[75] Allen M. Misconceptions in primary science. Berkshire, England, New York, N.Y: Open University Press; McGraw-Hill; 2010.

[76] Terwogt MM, Stegge H, Rieffe C. Children’s understanding of inherited resemblance: The case of two parents. International Journal of Behavioral Development. 2003;27(4):366–74. doi: 10.1080/01650250344000037

[77] Menendez D, Donovan AM, Mathiaparanam ON, Seitz V, Sabbagh NF, Klapper RE, et al. Deterministic or probabilistic: U.S. children’s beliefs about genetic inheritance 2023. 10.1111/cdev.14053 38169300

[78] Menendez D, Mathiaparanam ON, Seitz V, Liu D, Donovan AM, Kalish CW, et al. Like mother, like daughter: Adults’ judgments about genetic inheritance. J Exp Psychol Appl. 2023;29(1):63–77. doi: 10.1037/xap0000436 35834230

[79] Banet E, Ayuso E. Teaching genetics at secondary school: A strategy for teaching about the location of inheritance information. Sci Ed. 2000;84(3):313–51. doi: 10.1002/(sici)1098-237x(200005)84:3<313::aid-sce2>3.0.co;2-n

[80] Lewis J, Wood-Robinson C. Genes, chromosomes, cell division and inheritance - do students see any relationship? International Journal of Science Education. 2000;22(2):177–95. doi: 10.1080/095006900289949

[81] Lampert P, Scheuch M, Pany P, Müllner B, Kiehn M. Understanding students’ conceptions of plant reproduction to better teach plant biology in schools. Plants People Planet. 2019;1(3):248–60. doi: 10.1002/ppp3.52

[82] Stavy R, Wax N. Children’s Conceptions of Plants as Living Things. Human Development. 1989;32(2):88–94. doi: 10.1159/000276367

[83] Berti AE, Barbetta V. Conceptions about the origin of species of Italian 3rd, 4th, 5th, and 8th graders. In: García Madruga JA, Kohen R, Barrio Cd, editors. Construyendo mentes: Essays in honor of Juan Delval. Madrid: UNED - Universidad Nacional de Educación a Distancia; 2012. p. 337–341.

[84] Emmons N, Smith H, Kelemen D. Changing Minds With the Story of Adaptation: Strategies for Teaching Young Children About Natural Selection. Early Education and Development. 2016;27(8):1205–21. doi: 10.1080/10409289.2016.1169823

[85] Emmons N, Lees K, Kelemen D. Young children’s near and far transfer of the basic theory of natural selection: An analogical storybook intervention. J Res Sci Teach. 2017;55(3):321–47. doi: 10.1002/tea.21421

[86] Legare CH, Lane JD, Evans EM. Anthropomorphizing Science: How Does It Affect the Development of Evolutionary Concepts? Merrill-Palmer Quarterly. 2013;59(2):168. doi: 10.13110/merrpalmquar1982.59.2.0168

[87] Berti AE, Barbetta V, Toneatti L. Third-Graders’ Conceptions About the Origin of Species Before and After Instruction: an Exploratory Study. Int J of Sci and Math Educ. 2015;15(2):215–32. doi: 10.1007/s10763-015-9679-5

[88] Brown SA, Ronfard S, Kelemen D. Teaching natural selection in early elementary classrooms: can a storybook intervention reduce teleological misunderstandings? Evo Edu Outreach. 2020;13(1). doi: 10.1186/s12052-020-00127-7

[89] Ronfard S, Brown S, Doncaster E, Kelemen D. Inhibiting intuition: Scaffolding children’s theory construction about species evolution in the face of competing explanations. Cognition. 2021;211:104635. doi: 10.1016/j.cognition.2021.104635 33713876

[90] Shtulman A, Neal C, Lindquist G. Children’s Ability to Learn Evolutionary Explanations for Biological Adaptation. Early Education and Development. 2016;27(8):1222–36. doi: 10.1080/10409289.2016.1154418

[91] Evans EM. The emergence of beliefs about the origins of species in school-age children. Merrill-Palmer Quarterly. 2000;46:221–54.

[92] Kelemen D, Emmons NA, Seston Schillaci R, Ganea PA. Young children can be taught basic natural selection using a picture-storybook intervention. Psychol Sci. 2014;25(4):893–902. doi: 10.1177/0956797613516009 24503874

[93] Berti AE, Toneatti L, Rosati V. Children’s Conceptions About the Origin of Species: A Study of Italian Children’s Conceptions With and Without Instruction. Journal of the Learning Sciences. 2010;19(4):506–38. doi: 10.1080/10508406.2010.508027

[94] Samarapungavan A, Wiers RW. Children’s thoughts on the origin of species: A study of explanatory coherence. Cogn Sci. 1997;21:147–77.

[95] Shtulman A, Checa I. Parent-child conversations about evolution in the context of an interactive museum display. International Electronic Journal of Elementary Education. 2012;5:27–46.

[96] Tenenbaum HR, Hohenstein JM. Parent-child talk about the origins of living things. J Exp Child Psychol. 2016;150:314–29. doi: 10.1016/j.jecp.2016.06.007 27388483

[97] Frejd J, Stolpe K, Hultén M, Schönborn KJ. Making a fictitious animal: 6-7 year-old Swedish children’s meaning making about evolution during a modelling task. Journal of Biological Education. 2020;56(3):323–39. doi: 10.1080/00219266.2020.1799843

[98] Grether GF. Developing & Testing Curricula for Teaching Evolutionary Concepts at the Elementary School Level. The American Biology Teacher. 2021;83(2):96–103. doi: 10.1525/abt.2021.83.2.96

[99] Nadelson LS. Preservice Teacher Understanding and Vision of how to Teach Biological Evolution. Evo Edu Outreach. 2009;2(3):490–504. doi: 10.1007/s12052-008-0106-z

[100] Nadelson LS, Southerland SA. Development and Preliminary Evaluation of the Measure of Understanding of Macroevolution: Introducing the MUM. The Journal of Experimental Education. 2009;78(2):151–90. doi: 10.1080/00220970903292983

[101] Walker CM, Ganea PA, Gopnik A. Children’s causal learning from fiction: Assessing the proximity between real and fictional worlds. Proceedings of the Annual Meeting of the Cognitive Science Society. 2012;34.

[102] Heredia SC, Furtak EM, Morrison D. Exploring the influence of plant and animal item contexts on student response patterns to natural selection multiple choice items. Evo Edu Outreach. 2016;9(1). doi: 10.1186/s12052-016-0061-z

[103] Yorek N, Şahin M, Aydın H. Are animals ‘more alive’ than plants?: Animistic-anthropocentric construction of life concept. Eurasia Journal of Mathematics, Science & Technology Education. 2009;5:371–80.

[104] Scott J. Children as respondents: The challenge for quantitative methods. In: James A, Christensen PM, editors. Research with children: Perspectives and practices. 2nd ed. New York, NY: Routledge; 2008. p. 87–108.

[105] Afitska O, Heaton TJ. Mitigating the effect of language in the assessment of science: A study of English‐language learners in primary classrooms in the United Kingdom. Science Education. 2019;103(6):1396–422. doi: 10.1002/sce.21545

[106] Kang H, Thompson J, Windschitl M. Creating Opportunities for Students to Show What They Know: The Role of Scaffolding in Assessment Tasks. Sci Ed. 2014;98(4):674–704. doi: 10.1002/sce.21123

[107] Williams JM. Children and adolescents’ understandings of family resemblance: a study of naïve inheritance concepts. Br J Dev Psychol. 2012;30(Pt 2):225–52. doi: 10.1111/j.2044-835X.2011.02031.x 22550946

[108] Nehm RH, Schonfeld IS. Does Increasing Biology Teacher Knowledge of Evolution and the Nature of Science Lead to Greater Preference for the Teaching of Evolution in Schools? Journal of Science Teacher Education. 2007;18(5):699–723. doi: 10.1007/s10972-007-9062-7

[109] Springer K, Keil FC. On the development of biologically specific beliefs: the case of inheritance. Child Dev. 1989;60(3):637–48. doi: 10.2307/1130729 2737013

[110] Solomon GE, Johnson SC, Zaitchik D, Carey S. Like father, like son: young children’s understanding of how and why offspring resemble their parents. Child Dev. 1996;67(1):151–71. doi: 10.2307/1131693 8605825

[111] Hayes AF, Krippendorff K. Answering the Call for a Standard Reliability Measure for Coding Data. Communication Methods and Measures. 2007;1(1):77–89. doi: 10.1080/19312450709336664

[112] Gholz S. Boy who grew a forest: The true story of Jadav Payeng. Sleeping Bear Press; 2019.

[113] Koo TK, Li MY. A Guideline of Selecting and Reporting Intraclass Correlation Coefficients for Reliability Research. J Chiropr Med. 2016;15(2):155–63. doi: 10.1016/j.jcm.2016.02.012 27330520 PMC4913118

[114] Beniermann A, Moormann A, Fiedler D. Validity aspects in measuring evolution acceptance: Evidence from surveys of preservice biology teachers and creationists. J Res Sci Teach. 2022;60(6):1223–65. doi: 10.1002/tea.21830

[115] NGSS Lead States. Next Generation Science Standards. Washington, D.C.: National Academies Press; 2013.

[116] Osterhaus C, Lin X, Koerber S. Measuring scientific reasoning in kindergarten and elementary school: validating the Chinese version of the Science-K Inventory. Educ Res Policy Prac. 2023. doi: 10.1007/s10671-023-09332-9

[117] Weber AM, Leuchter M. Measuring preschool children’s knowledge of the principle of static equilibrium in the context of building blocks: Validation of a test instrument. Br J Educ Psychol. 2020;90 Suppl 1:50–74. doi: 10.1111/bjep.12304 31292951

[118] Seshan VE, Whiting K. clinfun: Clinical trial design and data analysis functions. Version 1.1.1.; 2023.

[119] Allaire JJ, Ellis P, Gandrud, Christopher, Kuo K, Lewis BW, et al. networkD3: D3 JavaScript network graphs from R. 2025.

[120] Borgers N, Hox JJ. Reliability of responses in questionnaire research with children. Fifth International Conference on Logic and Methodology. Cologne, Germany; 2000.

[121] Kelemen D, The Child Cognition Lab. How the piloses evolved skinny noses. 1st ed. Boston, MA: Tumblehome Learning; 2017.

[122] Kelemen D, The Child Cognition Lab. How the dormacks evolved longer backs. Boston, MA: Tumblehome Learning; 2018.

[123] Early DM, Iruka IU, Ritchie S, Barbarin OA, Winn D-MC, Crawford GM, et al. How do pre-kindergarteners spend their time? Gender, ethnicity, and income as predictors of experiences in pre-kindergarten classrooms. Early Childhood Research Quarterly. 2010;25(2):177–93. doi: 10.1016/j.ecresq.2009.10.003

[124] Bauer J-R, Booth AE. Exploring potential cognitive foundations of scientific literacy in preschoolers: Causal reasoning and executive function. Early Childhood Research Quarterly. 2019;46:275–84. doi: 10.1016/j.ecresq.2018.09.007

