# Supplementary material for: Introducing CACIE: Development of the first Conceptual Assessment of Children’s Ideas about Evolution
Source: PLoS One. 2025 Sep 3;20(9):e0331380. doi: 10.1371/journal.pone.0331380 (PMC12407416; doi:10.1371/journal.pone.0331380)
Supplement: S1 File — (DOCX) [file pone.0331380.s001.docx]

**CACIE items and categories**

**Table of Contents**

| **Principle** | **Key Concept** | | **Item** | **Page** |
| --- | --- | --- | --- | --- |
| Variation | Individual Variation | | V1A | 2 |
|  |  |  | V1B | 2 |
|  | Origin of Variation | | V2A | 2 |
|  |  |  | V2B | 3 |
|  | Differences in Fitness | | V3A | 3 |
|  |  |  | V3B | 3 |
| Inheritance | Reproduction | | I1A | 3 |
|  |  |  | I1B | 4 |
|  | Inheritance of Variation | | I2A | 3 |
|  |  |  | I2B | 4 |
| Selection | Limited Resources | | S1A | 2 |
|  |  |  | S1B | 2 |
|  | Differences in Reproduction and Survival Rate | | S2A | 5 |
|  |  |  | S2B | 5 |
|  | Changes in Population | | S3A | 5 |
|  |  |  | S3B | 5 |
|  | Speciation | Origin and extinction of species on Earth | S4A | 2 |
|  |  |  | S4B | 3 |
|  |  | Common Ancestry | S4C | 5 |
|  |  |  | S4D | 6 |

**Table 1.** Part One; Examples: Dandelion, Apple tree, Red fox, Brown-lipped snail.

| **Item** | **Question** | **S** | **Description** |
| --- | --- | --- | --- |
| V1A | Look. The [taxon] has [heritable trait]. What do you think? Do all [taxa] have [heritable trait]? ... Why do you think (not) all [taxa] have [heritable trait]? Do you think that all [taxa] are different from each other? | 0 | no variation |
|  |  |  | between-species variation |
|  |  | 1 | variation only due to:   - environment |
|  |  |  | - sex |
|  |  |  | - age |
|  |  |  | unsuitable explanation |
|  |  |  | no explanation |
|  |  | 2 | biological variation (e.g., "inherent uniqueness") |
| V1B | If we could look inside the [taxon], we would find that the [taxon] has [inner trait]. What do you think? Do all [taxa] have [inner trait]? ... Why do you think (not) all [taxa] have [inner trait]? | 0 | no variation |
|  |  |  | between-species variation |
|  |  | 1 | variation only due to:   - environment |
|  |  |  | - sex |
|  |  |  | - age |
|  |  |  | unsuitable explanation |
|  |  |  | no explanation |
|  |  | 2 | biological variation (e.g., "inherent uniqueness") |
| V2A | What do you think? Why do some [taxa] have [trait 1] and some [taxa] have [trait 2]? … | 0 | between-species variation |
|  |  |  | unsuitable answer |
|  |  | 1 | variation originates from:   - environment |
|  |  |  | - sex |
|  |  |  | - age |
|  |  | 2 | inter-parental variation |
|  |  |  | [or other random factors (such as mutations)] |
| S1A | What do you think? Is there always enough [resource] for all [taxa] out there? Even if there are a lot of [taxa]? ... What do you think? Why is that? | 0 | unlimited resources |
|  |  | 1 | limited resources   - unsuitable explanation |
|  |  |  | - no explanation |
|  |  | 2 | limited resources due to:   - external (e.g., climate/ location) |
|  |  |  | - internal (e.g., competition) |
| S1B | What do you think? Does it happen sometimes that one [taxon] has more food than another [taxon]? ... What do you think? How does it happen that one [taxon] has more food than another [taxon]? | 0 | equal distribution |
|  |  | 1 | unequal distribution:   - unsuitable explanation |
|  |  |  | - no explanation |
|  |  | 2 | unequal distribution due to external (e.g., climate/ location) or internal (e.g., competition) |
| S4A | What do you think? Have there always been [taxa] in the world? Or was there also a time when there were no [taxa]? ... What do you think? How come that there are [taxa] now? | 0 | has always existed |
|  |  | 1 | has NOT always existed:   - unsuitable explanation |
|  |  |  | - no explanation |
|  |  | 2 | has NOT always existed but decended from other species (through evolution) |
| S4B | What do you think? Will there forever be [taxa] in the world? | 0 | will exist forever |
|  |  | 1 | will NOT exist forever:   - unsuitable explanation |
|  |  |  | - no explanation |
|  |  | 2 | will NOT exist forever because:   - they will go extict |
|  |  |  | - they will evolve |
| V3A | [Animals/plants] with [a good sense of smell/long roots] are more likely to get [food/water]. What do you think? Do all [taxa] [have a good sense of smell/grow long roots]? | 0 | all have the same preconditions |
|  |  |  | between-species variation |
|  |  | 1 | different preconditions due to:   - environment |
|  |  |  | - sex |
|  |  |  | - age |
|  |  |  | - unsuitable explanation |
|  |  |  | - no explanation |
|  |  | 2 | different preconditions ("inherent uniqueness") |
| V3B | What do you think? What would happen to a [taxon] that wouldn’t have [a good sense of smell/long roots]?... Would it live as long as other [taxa]? Or would it live longer or die earlier? | 0 | no effect |
|  |  |  | unsuitable answer |
|  |  | 1 | affects the individual (e.g., less food) but could NOT lead to an earlier death (live the same / live longer) |
|  |  | 2 | affects the individual, which could lead to an earlier death |
| I1A | What do you think? Do [taxa] have a mother / father? ...  Else: What do you think? Where does a [taxon] come from? … | 0 | no biological parents |
|  |  |  | no member of the same species are involved (e.g., a seed from the store) |
|  |  | 1 | comes to life through one other individual of the same species but expresses that individuals cannot reproduce sexually (e.g., a seed from another apple (tree)) |
|  | if answer = seed: Where does the seed come from? / Does it take one [xxx] to get a seed? Or does it get two [xxx] to get a seed? |  |  |
|  |  | 2 | biological parents (sexual reproduction possible) |
| Skip the next two items when child scored -1 or did not give in answer in I1A | | | |
| I2A | Now I will show you some more pictures of other animals and plants. And I want you to tell me, which ones could be the parents of this [taxon] / from which ones this [taxon] could have come from. ...What do you think? Could THIS [taxon] be a parent? / Could the [taxon] have come from THIS [taxon]? ... How can you tell? Why did you choose this? | 0 | does not consider heritable traits (e.g., only:   - species or size/age) |
|  |  |  | - size/age |
|  |  | 1 | considers heritable traits but does not follow the logic of inheritance (e.g., interbreeding) |
|  |  | 2 | considers heritable traits and follows the logic of inheritance |
| V2B | What do you think? Does a [taxon] look different or the same as its parents? / than the plants/animals it came from?... Why can a [taxon] look different than its parents? / than the plants/animals it came from?… | 0 | no variation |
|  |  | 1 | variation only due to:   - environment |
|  |  |  | - sex |
|  |  |  | - age |
|  |  |  | unsuitable explanation |
|  |  |  | no explanation |
|  |  | 2 | variation between parents (inter-parental) |
|  |  |  | other genetic origin |
| I2B | What do you think? Can a [taxon] have siblings? Do all siblings of a [taxon] look the same? … What do you think? Why do siblings look the same / different from / resemble each other? | 0 | no variation |
|  |  | 1 | variation only due to:   - environment |
|  |  |  | - sex |
|  |  |  | - age |
|  |  |  | unsuitable explanation |
|  |  |  | no explanation |
|  |  | 2 | variation between parents (inter-parental) |
|  |  |  | other genetic origin |
| I1B | Imagine. If we would put a group of [taxa] onto an island by themselves with a lot of food and water. We leave them alone then check in with it many years later. What do you think? Would there still be the same number of [taxa]? Would there be more or less?... Why do you think that is? | 0 | population size stays the same |
|  |  | 1 | population size changes due to other factors than reproduction (due to:   - migration |
|  |  |  | - death |
|  |  |  | - other) |
|  | if grow: Do you mean they grow bigger? Or did they become more? | 2 | population size increases (through reproduction) |

**Table 2.** Selection Scenario; Examples: Eagle fern, Hooded crow.

| **Intro** | **Animal Example** | | | **Plant Example** | |
| --- | --- | --- | --- | --- | --- |
|  | Imagine. There is an island in the middle of the sea. On this island, ... | | | Imagine. There is an island in the middle of the sea. On this island, ... | |
|  | there live crows called hooded-crows. Let’s take a closer look at those crows. | | | there live ferns called eagle ferns. Let’s take a closer look at those ferns. | |
|  | There is one group of crows with larger beaks. With these big beaks they eat beetles. | | | There is one group of ferns that have poison in their leaves. Animals that try to eat the leaves get sick from it and quickly stop. | |
|  | And there is one group of crows with smaller beaks. With these smaller beaks they eat seeds. | | | And there is one group of ferns that do not have poison in their leaves. | |
|  | Now image, there is a disaster on the island and huge waves flood the part where the beetles have their burrows, and all of the beetles die. | | | Now imagine, a large swarm of grasshoppers flies to the island. Grasshoppers eat leaves and stems of plants. | |
|  | | | | | |
| **Item** | | **Question** | **S** | | **Description** |
| S2A | | What do you think? What happens now? ... Extended: What do you think? Do some of the [taxa] die? | 0 | | all are affected the same (no differences) |
|  |  |  | 1 | | effect on disadvantageous variants but they do NOT die |
|  |  |  |  |  | advantageous variants die |
|  |  | If yes without explanation: Is that true for one group? Or for both of them? | 2 | | death of individuals with disadvantageous traits |
| S2B | | What do you think? Will there be more [offspring] of one group of [taxa]? Why so? | 0 | | all are affected the same (no differences) |
|  |  |  | 1 | | more offspring of advantageous variants but:   - unsuitable explanation |
|  |  | If yes without explanation: Is that true for one group? Or for both of them? |  |  | - no explanation |
|  |  |  |  |  | disadvantageous variants have more offspring |
|  |  |  | 2 | | more offspring advantageous variants (because they were more likely to survive) |
| S3A | | In the beginning we had the same number of [taxa with advantageous trait] and [taxa with disadvantageous trait]. Now a few years go by. What do you think? Are there still as many [taxa with disadvantageous trait] as before? | 0 | | population size does NOT change |
|  |  |  | 1 | | population size changes through other factors than death |
|  |  |  | 2 | | less/no [taxa with disadvantageous trait] (because they are more likely to die) |
| S3B | | What do you think? Are there still as many [taxa with advantageous trait] as before? | 0 | | population size does NOT change |
|  |  |  | 1 | | population size changes through other factors than surviving and reproducing successfully |
|  |  |  | 2 | | more [taxa with advantageous trait] (because they are more likely to survive and reproduce) |
| S4C | | Look. This is a [taxon]. And here I have another picture of a [closely related taxon] and a [closely related taxon]. What do you think? How come these [animals/plants] look so much alike? | 0 | | no specific reason / unsuitable answer (e.g. all have black feathers / green leaves) |
|  |  |  | 1 | | they are all of the same kind (e.g., they are all [birds/crows/ferns]) |
|  |  |  | 2 | | they are related (through evolution) |
| **Item** | | **Question** | **S** | | **Description** |
| S4D | | Do you think a [taxon] and a [closely related taxon] could have [offspring] together? | 0 | | the individuals could reproduce |
|  |  |  | 1 | | the individuals could NOT reproduce (due to:   - no reason |
|  |  |  |  | | - unsuitable explanation) |
|  |  |  | 2 | | the individuals could NOT reproduce because the different species cannot interbreed |
