# Supplementary figures and images for: Introducing CACIE: Development of the first Conceptual Assessment of Children’s Ideas about Evolution
Source: PLoS One. 2025 Sep 3;20(9):e0331380. doi: 10.1371/journal.pone.0331380 (PMC12407416; doi:10.1371/journal.pone.0331380)

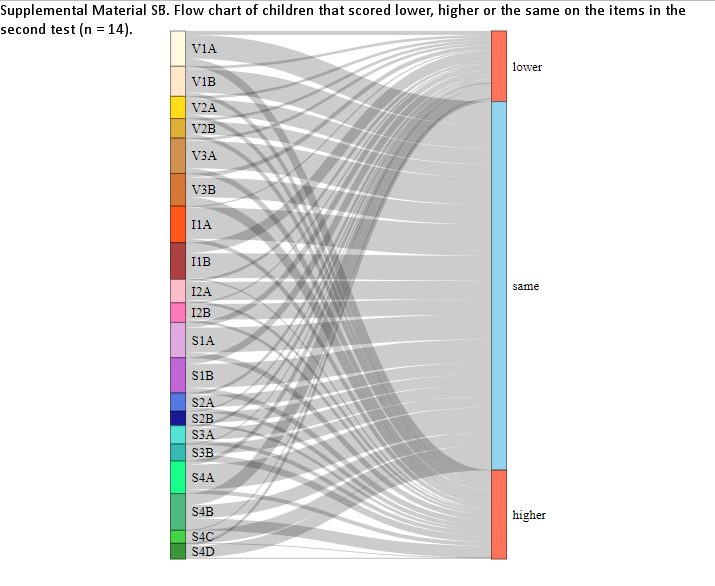

Supplement: S3 Fig — (PNG) [file pone.0331380.s003.png]
