# Supplementary material for: Introducing CACIE: Development of the first Conceptual Assessment of Children’s Ideas about Evolution
Source: PLoS One. 2025 Sep 3;20(9):e0331380. doi: 10.1371/journal.pone.0331380 (PMC12407416; doi:10.1371/journal.pone.0331380)
Supplement: S4 File — (DOCX) [file pone.0331380.s004.docx]

## Guidelines for Using the CACIE

If researchers find the CACIE to be a valuable tool for your data collection, it is essential for them to become familiar with the structure of the items and categorization levels. The next step involves preparing the devices to be used for data collection. Researchers should then choose the platform of choice to implement the survey, select the examples they want to use in the survey, and decide whether they should alternate, for example in a pre-post-design. The analyses conducted revealed no significant differences between animal and plant example species, with the exception of the items pertaining to sexual reproduction (I1A) and variation in beneficial traits (V3A). Consequently, depending on the research question, the exemplars could be alternated from pretest to posttest.

To ensure consistency in the interviewer's approach throughout the interviews, it is recommended that the interviewing person speaks through the survey several times to become accustomed to the questions and to think about how to help the children understand the questions, for example by pointing at the features that are referred to in the item. They should also consider how to respond to the children's answers without influencing their responses (i.e., through indicating that they said something right or wrong). While the children that we interviewed spoke, we listened with interest and gave verbal and non-verbal encouragement (e.g., through saying "m-hm”, “uh-huh" or through nodding). When we had gathered enough information to categorize the answer, we signaled to the children that we understood what they meant (e.g., "Okay.", "Ah, I see.", "Ah, okay, I get it."). Piloting the survey can also help prepare for the actual data collection. In the development of the CACIE, careful measures were taken to avoid intruding on children's ideas and to prevent any influence on their beliefs. As a result, items V2B and I2A are skipped when their questions conflict with the children's beliefs. However, this deliberate omission leads to missing data. Therefore, researchers using the CACIE should make informed choices on how to handle these missing values during statistical analyses prior to data collection. We also recommend interrating the interview in real time, on site. This approach enables the researchers to discuss conflicts right away. Our survey included a comment section, where the interrater can make short notes to support the discussion afterward or indicate why they rated an item a particular way. Before heading into the field, all interviewers and interraters should prepare by utilizing our training material to (1) prepare for the answers that children might give, (2) get an idea of the kind of follow-up questions that can help to categorize the children’s answers appropriately, and (3) discuss coding decisions beforehand.

If researchers plan to use a pre-post-design, it is recommended to provide an identifier for the participants to connect the data correctly. For our test-retest-design, we printed tickets with pictures of different species. The children chose a ticket and were told that these served as entry tickets for the interviews and reading session. The preschool teachers wrote down the children's names and animals and were further responsible for handing out the corresponding ticket to the children for each session in which we engaged with them. The teachers had no access to the collected data, and we had no information about the children’s names.
